# A novel unified Inception-U-Net hybrid gravitational optimization model (UIGO) incorporating automated medical image segmentation and feature selection for liver tumor detection

**DOI:** 10.1038/s41598-025-14333-0

**Published:** 2025-08-14

**Authors:** Tathagat Banerjee, Davinder Paul Singh, Pawandeep Kour, Debabrata Swain, Shubham Mahajan, Seifedine Kadry, Jungeun Kim

**Affiliations:** 1 Department of Computer Science & Engineering, IIT Patna, Patna, India; 2 Department of Computer Science & Engineering, Pandit Deendayal Energy University, Gandhinagar, Gujarat India; 3 Department of Chemistry, University of Kashmir, Srinagar, Jammu & Kashmir India; 4 Amity School of Engineering & Technology, Amity University Haryana, Gurgaon, India; 5 Department of Computer Science and Mathematics, Lebanese American University, Beirut, Lebanon; 6 Department of Computer Engineering, Inha University, Incheon, Republic of South Korea

**Keywords:** Liver tumor segmentation, Medical imaging, UIGO, Deep learning, Image segmentation, Machine learning, Diseases, Engineering

## Abstract

Segmenting liver tumors in medical imaging is pivotal for precise diagnosis, treatment, and evaluating therapy outcomes. Even with modern imaging technologies, fully automated segmentation systems have not overcome the challenge posed by the diversity in the shape, size, and texture of liver tumors. Such delays often hinder clinicians from making timely and accurate decisions. This study tries to resolve these issues with the development of UIGO. This new deep learning model merges U-Net and Inception networks, incorporating advanced feature selection and optimization strategies. The goals of UIGO include achieving high precision segmented results while maintaining optimal computational requirements for efficiency in real-world clinical use. Publicly available liver tumor segmentation datasets were used for testing the model: LiTS (Liver Tumor Segmentation Challenge), CHAOS (Combined Healthy Abdominal Organ Segmentation), and 3D-IRCADb1 (3D-IRCAD liver dataset). With various tumor shapes and sizes ranging across different imaging modalities such as CT and MRI, these datasets ensured comprehensive testing of UIGO’s performance in diverse clinical scenarios. The experimental outcomes show the effectiveness of UIGO with a segmentation accuracy of 99.93%, an AUC score of 99.89%, a Dice Coefficient of 0.997, and an IoU of 0.998. UIGO demonstrated higher performance than other contemporary liver tumor segmentation techniques, indicating the system’s ability to enhance clinician’s ability to deliver precise and prompt evaluations at a lower computational expense. This study underscores the effort towards advanced streamlined, dependable, and clinically useful devices for liver tumor segmentation in medical imaging.

## Introduction

### Motivation

Segmentation of both liver and tumors on CT scans is critical in medical imaging as it allows for accurate evaluation, diagnosis, therapy planning, and assessment after therapy. However, manual segmentation is both tedious and error-prone, increasing the need for automation to boost accuracy and efficiency^1^. The accuracy and performance of medical image segmentation have been greatly enhanced by deep learning techniques, especially… Deep Learning Techniques. U-Net and its cascade variants continue to be among the most popular architectures used for achieving optimization in segmentation results^2]– [3^.

In spite of these advancements, the accurate segmentation of liver tumors in CT images poses challenges because of the complex and low-contrast tumor shapes and their varying sizes in relation to surrounding tissues^4^. Hybrid CNN models combined with object-based post-processing have shown promise in tackling these problems^5^. Moreover, segmentation performance has been improved by the integration of classical approaches such as fuzzy c-means clustering in combination with deep learning algorithms^6^. Further refinement is still required within the cited research, especially considering problems posed by weakly defined tumor outlines and variable intensity in CT scan images^7,8^. Algorithms such as hidden Markov models as well as hybrid deep attention-aware networks are examples of semi-automated techniques that have been applied to enhance segmentation results^9^. As an example of such application, the AHCNet model is proficient in the detailed segmentation of liver cancers owing to its attention-based hybrid model design^10^. The CHAOS dataset has been instrumental in establishing benchmark standards for segmentation and has facilitated the development of reliable models for liver tumor segmentation^11^. Furthermore, sophisticated techniques such as resection maps have reinforced the need for precise segmentation in improving the quality of surgical intervention^12^. Enhancements in segmentation of liver tumors due to dynamic parameter tuning, contrast imaging, and advanced methods of interconnectivity have also been explored^13^. Sophisticated clustering combined with level-set methods has significantly improved the precision of segmentation not only in CT images, but also in images with contrast enhancement^14^. Systematic reviews on the liver segmentation techniques center deep learning blended with classical approaches as the most effective way to meet the tough challenges posed and resulting in clinically useful systems that improve patient outcomes^15^.

### Background

The malignancy involving liver cancer, especially hepatocellular carcinoma, remains one of the leading causes of cancer mortality which emphasizes the importance of planning detection and treatment procedures. Computed tomography and Magnetic Resonance Imaging have become indispensable for the diagnosis of liver tumors and evaluation of the effectiveness of the therapy conducted^16^. Accurate boundary depiction of the liver and the tumor is important for many clinical procedures including surgery, radiotherapy, and monitoring the disease^17^. The earlier segmentation approaches, such as sparse shape composition, have laid groundwork for liver tumor segmentation, but they struggle due to cancer diversity and intensity variations in CT images^18^. In fully convolutional networks (FCNs) and generative adversarial networks (GANs), deep learning methodologies have recently advanced the domain of automatic segmentation and achieved exceptional accuracy^19^. The development of hybrid models, especially those combining Fully Convolutional Networks (FCNs) and deformable models, improved segmentation precision by capitalizing on multiple methods^20^. Baselines like the Liver Tumor Segmentation (LiTS) challenge provide standardized datasets and assessment criteria, thus propelling progress in the area and supporting reproducibility^21^. Cross-modality adaptation techniques like 3D conditional GANs have been effective in addressing imaging heterogeneity, thus enhancing segmentation model generalizability^22^. In addition, 3D deeply supervised networks have advanced liver segmentation considerably by using volumetric data along with hierarchical information^23^. The evaluation of techniques for liver tumor segmentation in the LiTS challenge highlights once more the need for improvements in this area, as the approaches tried to date still struggle with adequately handling oddly shaped or low contrast tumors^24^. The combination of cascaded deep residual networks and convolutional neural networks has proven effective in improving the detection and segmentation accuracy of liver lesions^25^. Furthermore, the application of deep learning methods, especially convolutional neural networks, has streamlined the segmentation process and made it suitable for clinical practice^26^.

### Problem statement

As noted, the use of AI and ML methods in liver tumor mapping may present additional opportunities toward increasing diagnostic accuracy and efficiency, but concerns around dataset heterogeneity and imbalance are also significant. The presence of underrepresented tumor subclasses in the LiTS and CHAOS datasets tend to make model training more difficult which can result in suboptimal applicability in real-life scenarios. Moreover, imaging data pre-annotated by experts is also prone to many non-standardized differences, which significantly hampers the ability to train dependable AI systems. Solving these problems using effective preprocessing strategies or novel algorithmic approaches will help advance the state of the art and improve the clinical outcomes for patients. There is still much work to be done to create dependable, efficient, and clinically practical solutions for segmentation in image data, making further investment in this research direction necessary.

### Significance of the study (Objectives)

- This study proposes a novel hybrid approach by integrating the U-Net architecture with Inception modules, enhancing feature extraction capabilities for more accurate liver tumor segmentation.
- Utilizing a customized loss function that combines focal loss, Dice loss, and Hausdorff loss effectively addresses class imbalance issues and enhances segmentation performance, particularly in complex medical images.
- The introduction of the Gravitational Optimization (GO) algorithm for hyperparameter tuning improves the model’s efficiency and performance, demonstrating a novel approach to optimizing deep learning models in medical imaging.
- The study conducts comprehensive evaluations using multiple datasets (LiTS, 3DIRCADb1, and CHAOS), providing a thorough analysis of model performance across different scenarios, thereby validating the effectiveness of the proposed methods.

### Scope of study

The objectives of this study are to review more sophisticated techniques for the identification of liver tumor boundaries with the aid of deep learning techniques, such as the improvement of U-Net and Inception-based architectures through Gravitational Optimization (GO). The existing focal loss is going to be augmented with the Dice loss and the Hausdorff loss as a novel loss function for enhancing segmentation in the scenario of the imbalanced dataset. The study will also involve an analysis of the models’ performance over different clinical scenarios using benchmark datasets, which include LiTS, 3DIRCADb1, and CHAOS. Moreover, this study will explore the methods for hyperparameter tuning to improve both the model performance and speed in liver tumor diagnosis and treatment decision-making process, therefore, expanding the social contribution of academic research in the field of medical image analysis.

## Related works

### Liver tumor

Convolutional neural networks (CNNs) have given us unprecedented results on the precision of liver tumor segmentation using deep learning. Their best performance has been noted in detecting and segmenting liver cancers within radiological images. Some studies have focused on improving CNN explainability with clinical imaging features for better diagnostics and clinical relevance^27^. Deep learning is advancing rapidly, especially in the segmentation of liver lesions on CT scans, which has been the focus of considerable attention. These approaches automate the segmentation using CNN-based architectures, which require far less human interaction while maintaining high precision and accuracy^28^. This challenge of automatic identification of liver tumors has been tackled by multi-task CNNs, which improved the domain by performing lesion detection and segmentation simultaneously^29^. Fully convolutional networks (FCNs) set a landmark within the detection and segmentation of liver lesions due to their end-to-end training paradigm, which allows them to achieve better segmentation^30^. Their use in medical imaging, especially in radiology, increased with the ability of FCNs to perform numerous segmentation tasks, thereby expanding their applicability^31^. A new strategy based on three-dimensional fully convolutional neural networks (3D-FCNNs), which uses volumetric data for segmentation, holds great promise for precisely segmenting liver and tumors in CT scans. These models show superb effectiveness in gathering spatial context, which makes them exceptionally competent for complex medical imaging tasks, as in reference^32^.

### Need for medical image segmentation

The segmentation of medical images is vital in health care because it helps recognize anatomical features and pathological processes, which are fundamental in any diagnosis, treatment, and even tracking the progress of the disease. Extraction of liver and tumor regions from imaging techniques like CT and MRI is critical in the control and treatment of cancer in the liver and other liver disorders^33^. Medical image segmentation and processing have seen transformative advancements by integrating deep learning and attention-based architectures. Banerjee has made significant contributions across various clinical applications using domain-specific neural frameworks. For instance, the HHO-UNet-IAA model offers a novel glaucoma segmentation technique combining Harris Hawks Optimization with an inception-attention UNet structure. In thoracic organ segmentation, Resio-Inception U-Net has been employed alongside deep cluster recognition to extract distributed visual feature patterns^47^. Lung and cervical cancer detection have also benefited from advanced pyramidal attention networks such as DY-FSPAN^42^ and IMATX, offering explainable predictions suited for real-time clinical validation. In the realm of infectious diseases, attention-based models have been successfully applied for pneumonia^45,46^, malaria^43^, and Mycoplasma pneumoniae detection^44^. The development of EEG-based seizure detection using multi-domain deep feature fusion (ACE-SeizNet)^41^ and breast cancer classification via aggressiveness delineation (CICADA-UCX)^48^ further showcases the versatility of deep neural models in biomedical contexts. Collectively, these works demonstrate a growing trend towards interpretable, domain-adapted architectures that enhance diagnostic accuracy while addressing the complexity and variability inherent in medical imaging data. The variety of imaging techniques, patient anatomy, and the nature of the diseases make accurate segmentation exceedingly difficult. To address these problems, self-configuring deep learning approaches such as nnU-Net have been developed that are robust and flexible to biomedical image segmentation challenges. Multi-level deep learning approaches have been developed to improve the efficiency and accuracy of liver and tumor segmentation, leveraging the strengths of different architectures to address complicated segmentation problems^34^. Segmentation is important not only for the exhaustive training of deep learning models, but also for refining pre-trained models wherein updating such neural networks has proven beneficial to aligning them with specific tasks in medical imaging, improving performance while reducing processing resources needed^35^. Alongside these advancements, incorporating traditional techniques such as graph optimization with deep learning U-Net models has led to improved accuracy in the segmentation of complex liver tumors^36^. The development of domain adaptation methods has brought attention to cross-modality segmentation, where problems due to differences in imaging modality and acquisition techniques have been tackled using unsupervised approaches with synthetic or generative adversarial networks^37^.

### Convolutional neural networks for image segmentation

The Convolutional Neural Networks (CNNs) have transformed the idea of image segmentation with impressive improvements in the application of medical image analysis in relation to liver tumor detection. The U-net architecture is one of the oldest architectures in the CNN-based segmentation method. U-Net follows the fully convolutional encoder-decoder architecture that passes context through the main down-sampling phases while preserving spatial relation via the main up-sampling stages. This type of architecture has been widely employed in the liver tumor segmentation problems because it addresses some of the usual difficulties of medical image analysis, including limited datasets. Because of this, it is quite ideal when it comes to mapping an image at the pixel level, which is especially important in outlining the tumor margins against an often-heterogeneous background.

**Fig. 1:**
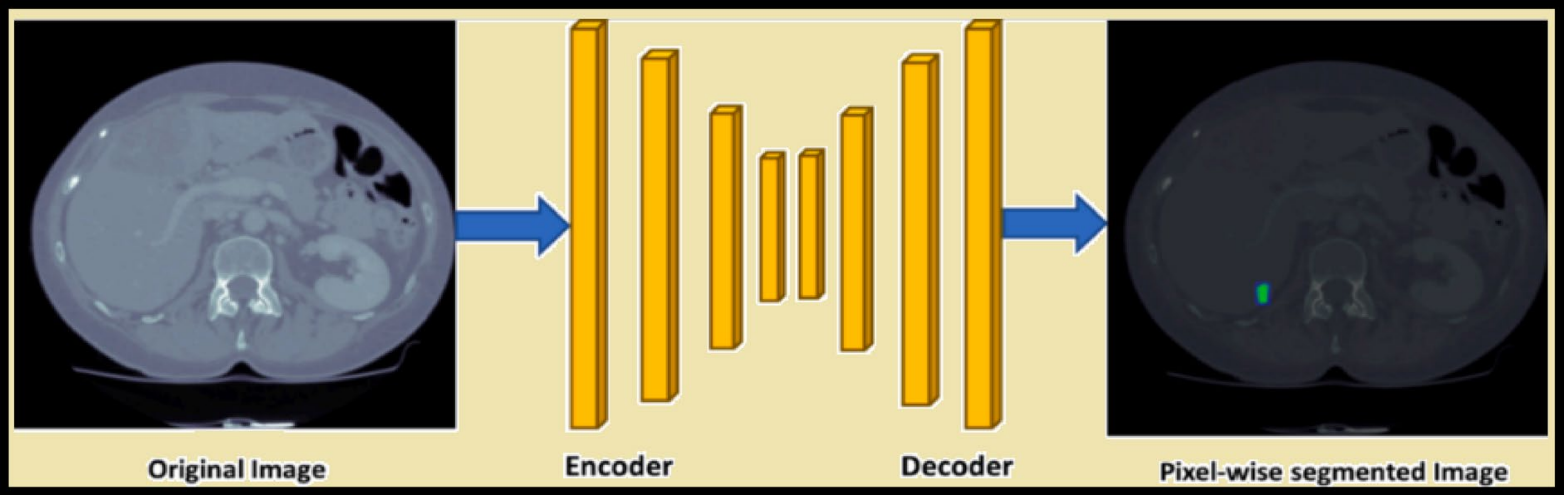
Liver Tumor Segmentation.

In the recent past, there were some updates and enhancements to the typical U-Net architecture as described below. For example, attention mechanisms have boosted feature representation and increased the correctness of experimental segmentation. U-Net-based model (as shown in Fig. 1 Liver Tumor Segmentation) for attention utilize attention gates into the U-Net structure that helps to calculate the weight for the feature maps, which plays an important role in distinguishing the tumor from healthy liver tissue.

### Liver tumor segmentation

Liver tumor segmentation is a core component of medical image analysis and directly affects the treatment plan and the resultant patient prognosis. The correct segmentation will help define the tumor’s exact location, size, and edges, which must be used in most operations and radiation therapy. As the shape of the liver and the presentation of the tumor are variable due to its structure and heterogeneity, this results in time-consuming and subjective manual segmentation. Therefore, the focus has been shifted to the automatic segmentation techniques based on deep learning models, including CNNs. New developments in deep learning have opened the way to complex models capable of segmenting liver solid tumors against different imaging techniques, such as CT, MRI, and ultrasound^38,39^. An incredible array of such imaging techniques complicates the work, as each modality offers contrast and resolution characteristics that affect the visibility of a tumor. For example, the CT scan is preferred for high spatial resolution and fast image acquisition for tumor detection. In contrast, MRI is chosen for superior soft tissue contrast required for distinguishing between tumor types. With the emergence of specific datasets, like the Liver Tumor Segmentation (LiTS), a fundamental role has been played in training and evaluating segmentation algorithms. Such challenges characteristic of this LiTS dataset, such as containing annotated CT scans with diverse tumor presentations, help train CNNs with the intent of generalizing their capabilities to other tumor sizes and configurations. It has given the research community a checking dataset to compare multiple models, and the possibility of new ideas on best segmentation has floated around due to this freely shared resource. Several deep learning methods have been found to give remarkable outcomes for liver tumor segmentation. For instance, integrating the CNN networks with other architectures, including RNNs or graph-based models, has been identified to boost the segmentation capability of the adopted machine learning architectures. These combined architectures use the capabilities of each of the above neural networks to learn spatial correlations that are presented within images.

**Table 1:** Literature review: comprehensive description.

Reference	Dataset Used	Techniques	Metrics
Aghamohammadi et al. (2021) A Two-Path CNN for Liver and Tumor Segmentation	LiTS 201	Two-Path CNN	Dice Score: 0.87, Precision: 0.88, Recall: 0.85
Albishri et al. (2019) Cascaded U-Net Models for Automatic Liver Tumor Segmentation	3D-IRCADb1	Cascaded U-Net Models	Dice Score: 0.83, Jaccard Index: 0.76
Anter & Hassenian (2019) Fuzzy c-means Clustering for Liver Tumor Segmentation	LiTS 201	Fuzzy C-Means Clustering	Dice Score: 0.80, Sensitivity: 0.82
Chlebus et al. (2018) Automatic Liver Tumor Segmentation in CT	LiTS 201	Fully Convolutional Neural Networks	Dice Score: 0.85, Accuracy: 0.90
Fan et al. (2020) MA-Net: Multi-scale Attention Network for Liver Tumor Segmentation	LiTS 201	Multi-scale Attention Network	Dice Score: 0.89, Precision: 0.91
Jiang et al. (2019) AHCNet: Attention Hybrid Convolutional Network for Liver Tumor Segmentation	LiTS 201	Attention Hybrid CNN	Dice Score: 0.86, F1 Score: 0.84
Kaur & Kaur (2024) Liver Tumor Segmentation Using the CHAOS Dataset	CHAOS	CNN, Transfer Learning	Dice Score: 0.81, Jaccard Index: 0.77
Zhang et al. (2012) Sparse Shape Composition for Liver Tumor Segmentation in CT Images	LiTS 201	Sparse Shape Composition	Dice Score: 0.82, Sensitivity: 0.79

In addition, the Table 1 Literature Review: Comprehensive description, showcases techniques, including CRF, that can be applied for the local post-processing aiming at enhancing the quality of the edges of the segmented areas and their perfect matching to the real contours of tumor manifestations. This step is essential in environments with a high degree of accuracy because all measurements are preferably accurate to the nearest millimeter. Notwithstanding such progress, some issues are still unresolved, especially regarding how segmentation algorithms operate in the context of real clinical data that can contain noise, artifacts, or variability in, for instance, image acquisition parameters. To mitigate these challenges, future research investigations plan to employ ensemble learning approaches that consider a set of models to generalize performance and self-supervision learning methods that help minimize the amount of annotated data required.

### Datasets and works toward liver tumor segmentation

The reason for developing accurate liver tumor segmentation algorithms is that many large annotated datasets are available. The LiTS (Liver Tumor Segmentation) benchmark has been one of the most popular benchmarks for liver tumor segmentation. This dataset includes abdominal CT volumes with annotations of liver and liver tumors to allow obtaining a strong ground truth. Work based on data of this kind has inspired such segmentation models as RA-UNet, which is based on paying attention to FCNs and applies to the segmentation of liver and tumors^9^.

Furthermore, the CT images with multiphase contrast enhancement are available in the CHAOS dataset, which has been applied to segment liver tumours, revealing the importance of multimodal images in advancing segmentation results^11^. Other valuable approaches integrating FCNs with NMF-based deformable models^19^ and employing cascaded U-Net models^2^ have also enhanced LSTM for liver tumor segmentation.

### Research question

1. How can segmentation models be designed to enhance generalizability across diverse clinical settings and patient demographics, thereby improving their practical applicability in liver tumor segmentation?
2. What strategies can be implemented to address class imbalance in medical imaging, ensuring that segmentation models perform optimally in accurately identifying tumor instances despite their rarity compared to healthy tissue?
3. How can systematic approaches to hyperparameter optimization be developed and integrated into deep learning architectures to improve the accuracy and efficiency of liver tumor segmentation methods, while ensuring comprehensive evaluations across multiple datasets?

## Database description and preprocessing pipeline (DDPP)

### Dataset description

#### Liver tumor segmentation (LiTS 201)

**Fig. 2:**
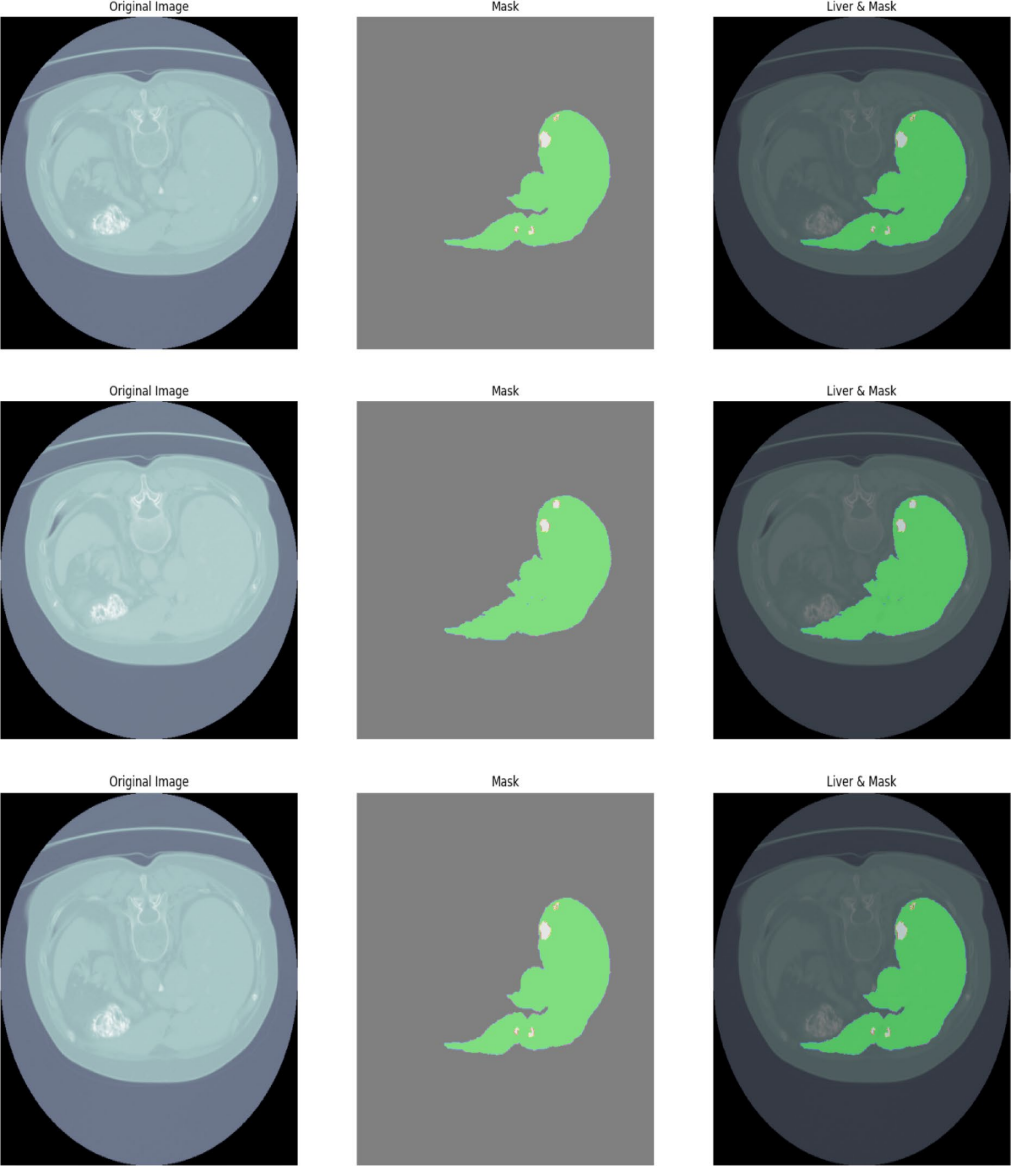
LiTS 201 Sample Image.

Liver Tumor Segmentation (LiTS) is another public dataset tirelessly used for training and evaluation of the segmentation models commonly sourced from the LiTS 201 dataset^40^ including 201 images from different patients with different liver diseases of various sizes and shapes of tumor lesions as well as the CT images of the corresponding healthy people as shown in Fig. 2. The ground truth is given for both liver and tumor regions, making it possible to employ the dataset for multi-organ segmentation. In the present study, scans are acquired from several hospitals, implicating significant inter-site variability in imaging parameters and scanner manufacturers. This variation provides a range of data that can be extremely useful to build effective segmentation strategies. LiTS 201 has a moderate number of livers and tumors, and therefore, it is suitable for deep learning schemes where a large set of sample data is required for training and testing.

#### 3D-IRCADb1

**Fig. 3:**
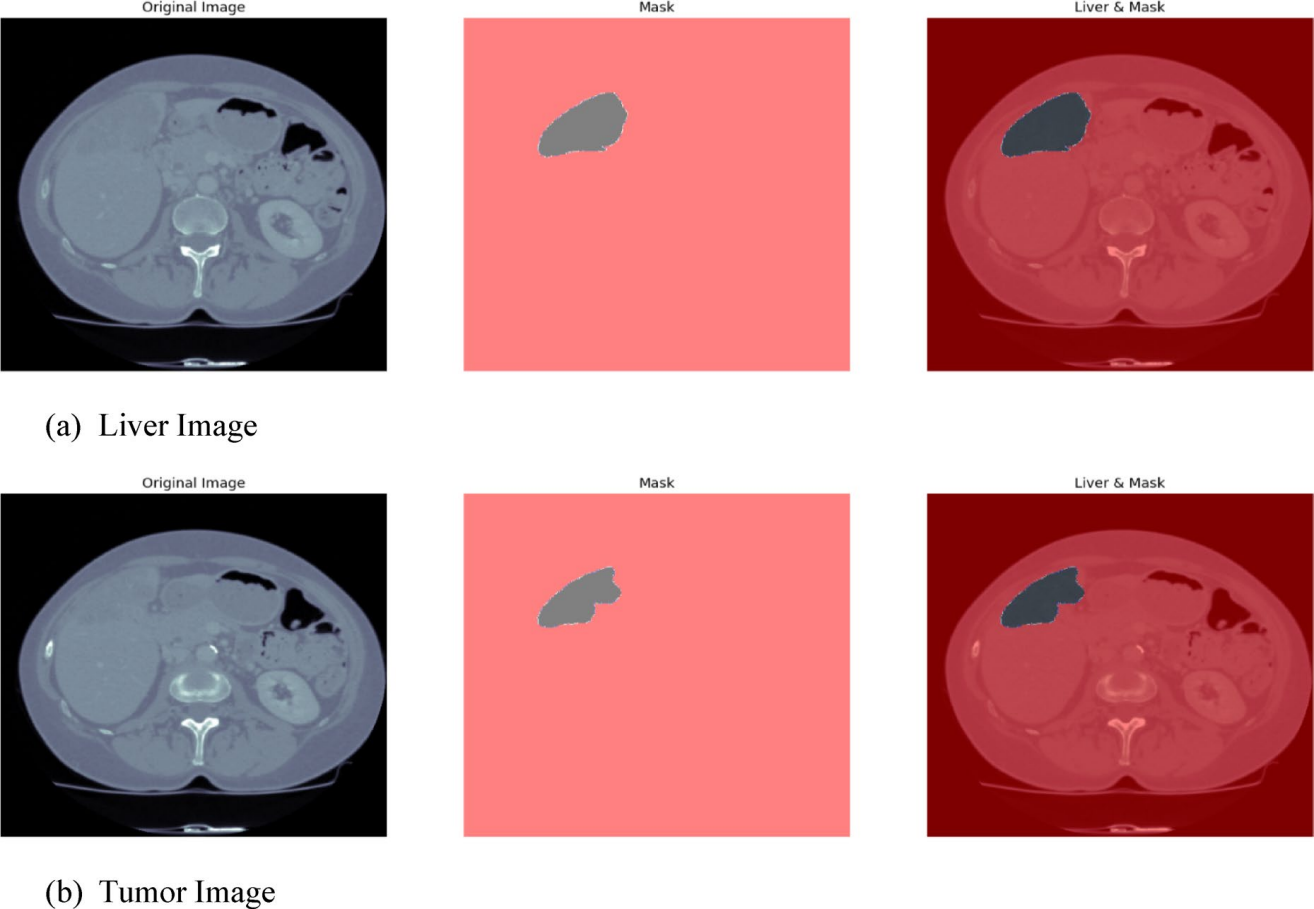
3D-IRCADb1 Sample Image.

The 3D-IRCADb1 contains the 3D CT of abdominal regions, specifically liver, liver tumor, and organ at risk (OAR) segmentation as shown in Fig. 3. It consists of CT scans from twenty patients who have liver pathologies; nineteen of the patients exhibit multiplicity of liver lesions. The liver, liver lesions, and the surrounding organs have also been manually annotated in the dataset, which makes the dataset very useful for both segmentation and multi-organ detection. The scans are high resolution and are given in 3D format, enabling volumetric analysis and organ segmentation. It also generates different annotations for surrounding organs such as kidneys, lungs, and the stomach, which makes it preferred in the segmentation of OAR in radiotherapy planning scans.

#### CHAOS

**Fig. 4:**
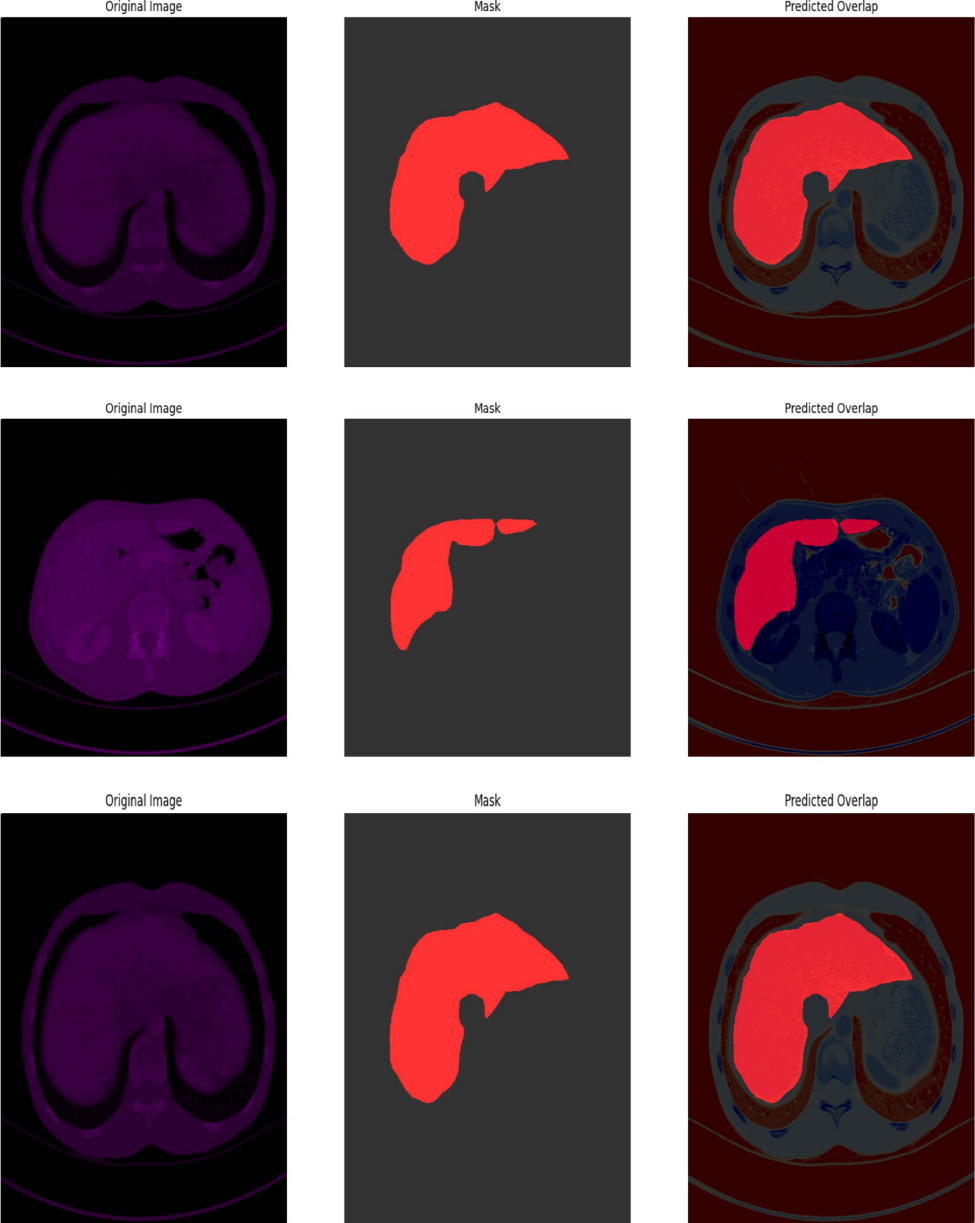
CHAOS Sample Image.

The CHAOS dataset contains images in the form of combinations of healthy CT and MRI scans, where each organ is segmented. The dataset is therefore intended to provide segmentation models with different organs under different imaging scenarios. Fourteen patients from the CHAOS database have CT scans and annotations of the liver, as shown in Fig. 4.

**Table 2:** Dataset Summary.

Dataset Name	Number of Patients	Imaging Modality	Key Annotations	Number of Liver Tumors	Other Organs Annotated	Application
LiTS 201	201	CT	Liver and Tumor	Various sizes and shapes	No	Liver and liver tumor segmentation
3D-IRCADb1	20	CT	Liver, Tumors, Organs	Multiple lesions	Kidneys, Lungs, Stomach	Multi-organ segmentation
CHAOS	40	CT, MRI	Healthy liver, other organs	N/A	Kidneys, Spleen	Healthy organ segmentation

Table 2 Dataset Summary Summarize the three most used datasets in liver and multi-organ segmentation studies. The LiTS 201 comprises 201 patients’ CT scans with comprehensive annotations based on liver and tumors of different sizes, so this competition is aimed explicitly at liver and tumor segmentation. The 3D-IRCADb1 contains 20 patients and uses CT images to segment the liver, tumor, and several other internal organs like kidneys, lungs, and stomach, where ground truth data is provided. Finally, CHAOS has both CT and MRI scans of 40 patients, mainly for healthy liver and kidney/spleen segmentation tasks, available. Different datasets are designed to meet specific segmentation tasks. The LiTS is primarily concerned with liver tumors; 3D-IRCADb1 provides the multi-organ dataset, while the CHAOS covers the healthy liver and organs in various imaging modalities.

Table 3 Dataset Image Use Showcases the training, validation, and testing sequences in the ratio of 70%, 20% and 10% respectively.

**Table 3:** Dataset image Use.

Dataset	Class	Training Instances	Validation Instances	Testing Instances	Total Instances
LiTS	Liver	141	40	20	201
LiTS	Tumor	141	40	20	201
3DIRCADb1	Liver	1860	532	266	2658
3DIRCADb1	Tumor	1860	532	266	2658
3DIRCADb1	Liver	1531	437	219	2187
3DIRCADb1	Tumor	1531	437	219	2187
CHAOS	Liver	4485	1281	641	6407
CHAOS	Tumor	4485	1281	641	6407

#### Preprocessing pipeline

Preprocessing is significant in preparing medical images for analysis and segmentation, especially in tasks that involve the detection of liver and tumors. Another procedure applied to improve pixel quality and comparability of pictures among the databases is normalization and standards. All data pre-processing methods return scaled pixel values to the value in the range [0, 1], which helps achieve a better degree of uniformity of the data, making the deep learning model converge faster. On the other hand, standardization changes the data distribution to have its mean equal to zero and its standard deviation equal to one, which is particularly useful when working with datasets of different intensities. Equations 1 and 2 allow grey scaling and normalization on the image.

1 $$\:{i}^{{\prime\:}}=\frac{i-{i}_{min}\cdot\:}{{i}_{max}\:\:-\:{i}_{min}}$$

2 $$\:{i}_{GREY}=0.299\:\left({i}_{R}^{{\prime\:}}\right)+0.587\left({i}_{G}^{{\prime\:}}\right)+0.114\left({i}_{B}^{{\prime\:}}\right)$$

Our method uses bilateral filtering and Gaussian blurring for the denoising process; this helps to remove the noise while it retains edges, which are essential for structure and anatomy in medical images. Bilateral filtering contrasts both spatial relationships and the level of intensities in the picture, which involves the smoothing of the picture without distorting the edges, unlike Gaussian blurring, which applies a kernel to put into practice a weighted average of intensities of a Gaussian blur, which is helpful for the reduction of high-frequency noise. In Eqs. 4 and 5, where σ_s_ ​ controls the spatial extent of the filter, σ_r_​ determines the range of intensity values considered. And Eq. 3 Showcases the bilateral function.

3 $$\:Bilateral\:Filtering\:\left(I\right)\left({\varDelta\:}_{x,y}\right)=\left(\frac{1}{{W}_{xy}}\right)\sum_{\left(a,b\right)\in\:{N}_{ab}}^{{N}_{ab}}I\left({\varDelta\:}_{a,b}\right)\:.\:\:{W}_{s}\left({\varDelta\:}_{a,b},\:{\varDelta\:}_{x,y}\right)\:.\:{W}_{r}({\varDelta\:}_{a,b},\:{\varDelta\:}_{x,y})$$

4 $$\:{W}_{s\:}\:\left({\varDelta\:}_{a,b},\:{\varDelta\:}_{x,y}\right)\:or\:Spatial\:kernel=\:{e}^{\frac{(-{\left|\right|\:{\varDelta\:}_{a,b},\:{\varDelta\:}_{x,y}\:\left|\right|}^{2}\:)}{2{\sigma\:}_{s}}}\:$$

5 $$\:{W}_{r\:}\:\left({\varDelta\:}_{a,b},\:{\varDelta\:}_{x,y}\right)\:or\:Radial\:kernel=\:{e}^{\frac{(-{\left|\right|\:{\varDelta\:}_{a,b},\:{\varDelta\:}_{x,y}\:\left|\right|}^{2}\:)}{2{\sigma\:}_{r}}}\:$$

In addition, augmentation methods have the foremost impact of increasing geometric ‘sturdiness’ or generating further data for artificially training the model. Rotated, flipped, scaled, and translated images preserve similar features of the prototypes, making it possible for the model to learn better generalizations and vagueness and minimize cases of overfitting. Altogether, these preprocessing methods work synergistically and refine the input data, promote improvements in the model, and drive better liver and tumor segmentation.

## UIGO – Evolutionary enhancement of Unet inception architecture

**Fig. 5:**
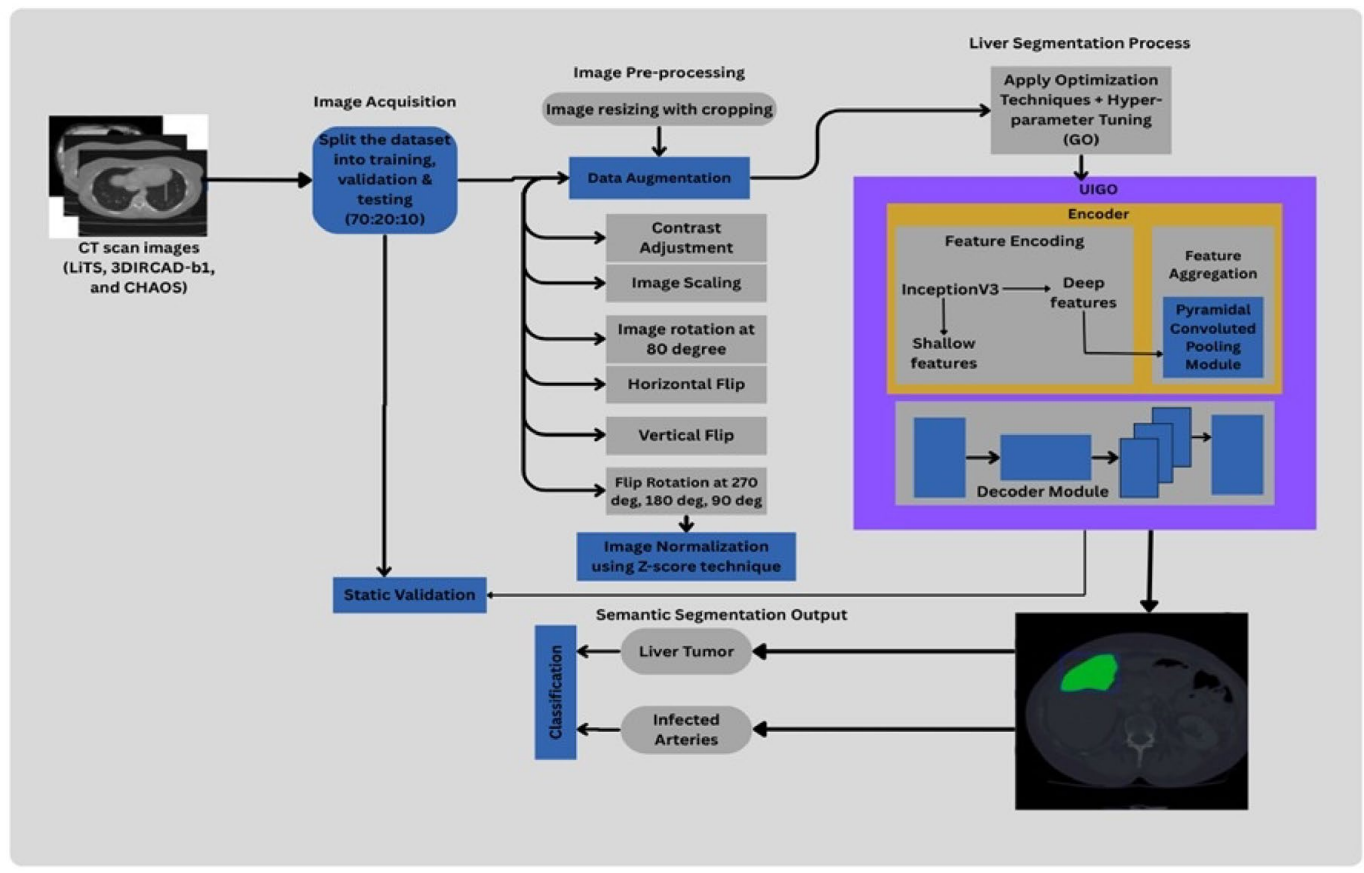
Proposed UIGO-Based Liver Tumor Segmentation Framework with Pre-processing, Inception-Enhanced Encoding, and Gravitational Optimization.

The liver tumor segmentation pipeline is illustrated by Fig. 5 Presents a comprehensive automated medical image analysis framework using the proposed UIGO model. It begins with acquiring CT scan images from three benchmark datasets—LiTS, 3DIRCADb1, and CHAOS—which are then partitioned into training, validation, and testing sets in a 70:20:10 ratio to ensure balanced evaluation. Pre-processing involves image resizing with cropping, followed by extensive data augmentation techniques including contrast adjustment, image scaling, multi-angle rotations (80°, 90°, 180°, 270°), and horizontal and vertical flips to enhance generalizability. Subsequently, Z-score normalization is applied to standardize pixel intensity values across all images. The framework’s core is the UIGO segmentation model, which integrates the U-Net encoder-decoder structure with InceptionV3 blocks for multi-scale feature extraction. Shallow and deep features are processed through a Pyramidal Convoluted Pooling Module for rich spatial-contextual representation. These features are aggregated and decoded to generate precise liver tumor segmentation maps. Gravitational Optimization (GO) is incorporated for efficient hyperparameter tuning, improving model accuracy and convergence. The final segmentation output is used to classify liver tumors and infected arteries, with static validation ensuring the reliability of predictions. This pipeline offers a robust, optimized, and interpretable solution for liver tumor segmentation in clinical imaging scenarios.

The proposed model consists of a two-stage modular pipeline that sequentially integrates segmentation and classification, not as a multi-task model. The first stage performs semantic segmentation using an Inception-ResNet-based U-Net variant. In contrast, the second stage conducts classification based on features extracted from the segmented output using Gravitational Optimization-enhanced LightGBM.

**Segmentation Module (Stage 1)**:

As depicted in Fig-7 The segmentation architecture is an enhanced U-Net that employs:

- Inception-ResNet backbone blocks for multi-scale feature encoding (orange blocks in Fig. 2),
- Levelled Bottlenecks (blue blocks in Fig-8) for gradient flow and feature refinement,
- Skip connections with residual aggregation to preserve spatial information,
- Historical Stamp Bypassing (triangular modules in Fig. 1) to facilitate information retention across layers,
- Input resolution: 512 × 512, with progressive down sampling till 32 × 32, and symmetrical upsampling in the decoder path.

Each convolutional layer in the encoder-decoder pipeline is set with kernel size = 3 × 3, stride = 1, followed by Batch Normalization, ReLU activation, and Dropout (rate = 0.3). The segmentation output is refined using Conv2D (1 × 1) with sigmoid activation to produce binary masks.

**Fig. 6:**
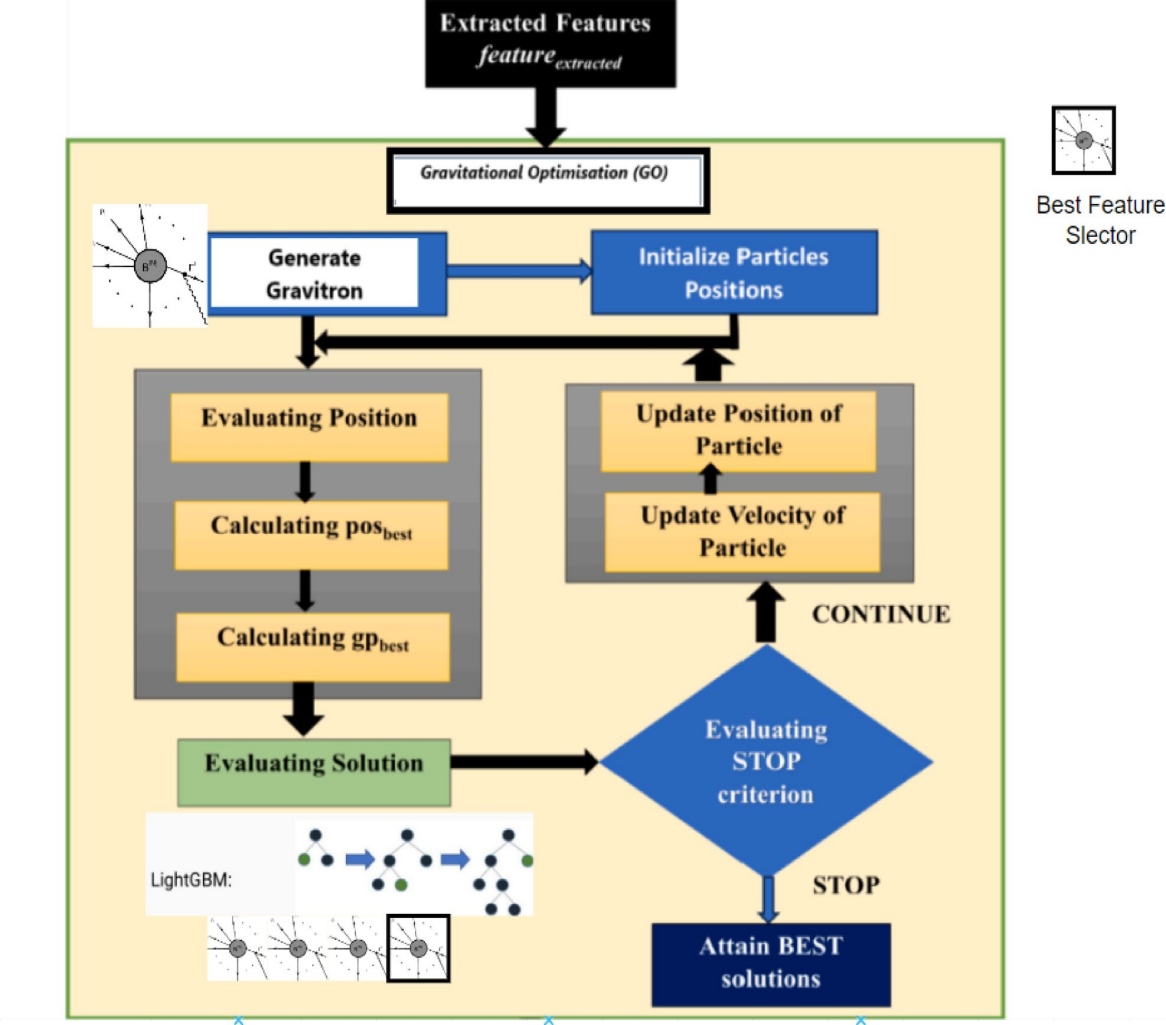
Gravitational Optimization.

**Feature Extraction and Classification (Stage 2)**:

The segmented output is passed into the second module for classification:

- Extracted features are pooled and passed to a Gravitational Optimization (GO) framework (Fig. 6) for best feature subset selection.
- The GO module initializes a swarm of particles (candidate feature subsets), evaluates fitness based on segmentation consistency and class separability, and iteratively updates the position and velocity of particles to converge on the optimal set.
- The best-selected features are finally classified using a LightGBM model, chosen for its efficiency and high performance in structured tabular data.

This architecture ensures that segmentation improves downstream classification by acting as a preprocessing stage, enabling more informative and localized feature selection. The core architectural enhancement in the UIGO model is embedding Inception modules into the encoder branch of the U-Net framework. These modules allow the model to extract features across multiple spatial resolutions by applying parallel convolutions of kernel sizes within each block. This design captures local textures and global contextual structures crucial for segmenting medical features like liver tumors, which often present with varying morphologies and indistinct boundaries. By fusing these multi-scale features early in the encoding process, UIGO gains a richer and more discriminative representation of anatomical regions.

### Gravitational optimization (GO)

To optimize the UIGO network, we employed the Gravitational Optimization (GO) algorithm for hyperparameter tuning, including learning rate, convolutional filter size, dropout ratio, and batch size. GO is a population-based metaheuristic inspired by Newtonian gravity, where candidate solutions are considered masses that attract each other proportionally to their performance (fitness). Unlike grid search, which suffers from the curse of dimensionality due to exhaustive evaluation across fixed parameter grids, or random search, which explores the space without directionality, GO dynamically guides the search process toward promising regions using fitness-informed updates. This makes it particularly suitable for tuning deep neural networks, where the hyperparameter landscape is often high-dimensional and non-convex. In our experiments, GO demonstrated faster convergence and better generalization performance than grid and random search, requiring fewer iterations to identify optimal configurations. This efficiency is attributed to its exploitation–exploration balance and adaptive learning behaviour.

### Background

Gravitational Optimization (GO) is an algorithm with a background in classical mechanics, specifically Newtonian gravitational law. The algorithm utilized in the process is inspired by the astronomical system wherein greater objects acted on lesser objects with more power. This analogy provides the basis for building an optimization method that emulates these processes.

In optimization, standard algorithms, e.g., gradient descent and genetic algorithms, have also been used to solve different problems. However, the issues and solutions associated with these methods are subject to local optima and the setting of many parameters. As such, there is an approach of exploring and exploiting simultaneously using gravitational interactions, which GO uses to solve these challenges.

While GO is a newly developed optimization algorithm, it was developed based on the laws of gravitation in physics. These concepts, including mass and gravitational attraction, are used to search and organize search spaces and therefore serve as an effective tool for optimizing several applications, such as deep learning in model training. GO uses a rather unconventional approach to the search for a solution by mimicking planetary systems governed by gravity.

In the case of improving deep learning architectures, GO works through the definition of the candidate solutions as if they were mass particles in several dimensions in the search space. The position of each particle is, in fact, a feasible solution for the optimization problem. The algorithm changes the particles’ positions using the particles’ mass, distance from other particles, and gravitational force of massive particles. It provides the ability to efficiently initiate the algorithm to search around the requested space and end in the optimal solutions. One of the significant strengths of GO is the organization’s capacity to juggle exploration and exploitation. In the first iterations of the optimization process, it guides the search towards greater distance from the current solution, which means that it has a higher ability to explore more of the search space. Over time, GO changes from exploring the search space to exploiting the area around the best solutions discovered. This adaptive behavior improves the algorithm’s performance, as far as it is concerned with configurations of deep learning systems. This makes GO easy to implement in many deep learning frameworks, thus making it flexible in many tasks. In the problem of liver tumor segmentation, GO enables optimization of hyperparameters ranging from learning rates, regularization factors, and the architecture of the neural networks, enhancing the possibility of higher segmentation accuracy and shorter times to converge during the training process. In general, the use of GO in conjunction with U-Net + Inception architectures, in this case, offers a vital path for improving the targeting of the biomedical image segmentation model. Combining both algorithms to increase the number of new features, architecture, and hyperparameters for segmenting is possible.

### Algorithmic details

The GO algorithm (Algorithm 1) entails a step known as the Gravitational Optimization Steps that help to optimize the process. First, the population of particles is randomly distributed throughout the Search Space, which is a possible solution to the problem. Every particle has an objective function value that corresponds to the quality of the solution that it advises. It uses an iterative updating approach in which every particle changes its position depending upon its velocity and the gravitational forces of other particles. Through the update mechanism, the new velocity and position for each particle are determined by the gravitational force. The velocity update considers the previous velocity and attraction towards the global best position, the international best. Figure 6: Gravitational Optimization showcases the workflow of GO.

**Algorithm 1:**
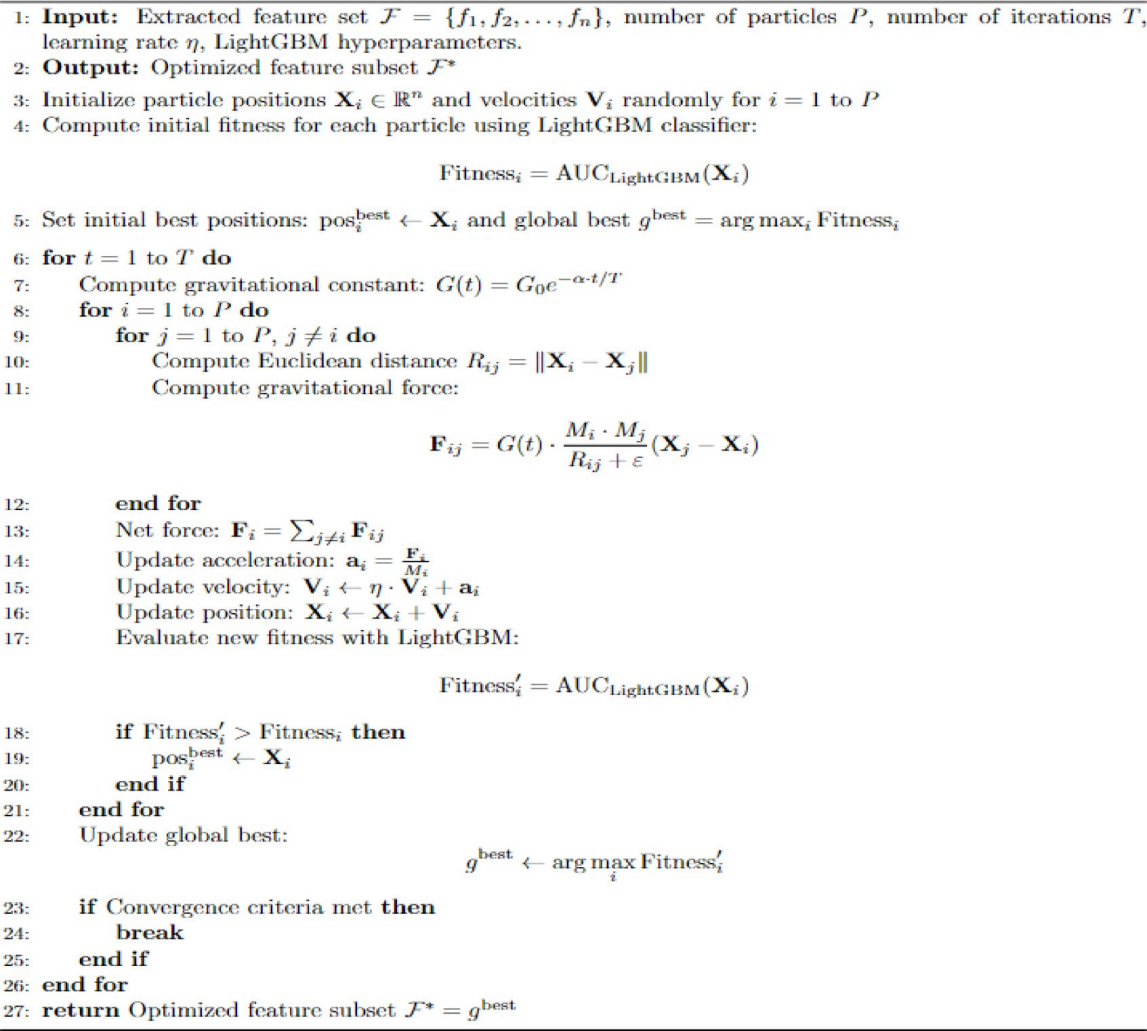
Gravitational Optimization (GO) with LightGBM discriminative evaluation.

#### Loss function

As applied for liver tumor segmentation within the U-Net + Inception framework, the Gravitational Optimization (GO) is used to adapt the model to achieve the least amount of defined loss function. The loss function is critical in guiding the training process, and in this case, it is a combination of three distinct loss metrics: It includes three losses, such as focal loss, Dice loss, and Hausdorff loss. The volume of samples being ignored in the minority class helps to enhance the result, especially in medical visual imaging, where the classes are imbalanced.

##### Focal loss (27%)

Focal loss is developed in a way that pays much attention to complex examples or samples to overcome the class imbalance problem. It underemphasizes the proportion of well-classified examples, and thus enhances the model’s ability to focus on complex cases such as small or less developed tumors in hepatic scans. The formula for focal loss is shown by Eq. 6.

6 $$\:f=-{\alpha\:}_{t}{\left(1-{P}_{t}\right)}^{\gamma\:}{log}\left({P}_{t}\right)$$

##### Dice loss (69%)

Dice loss is favorable in segmentation since it computes the similarity between the predicted segmentation map and the ground truth. It is defined by Eq. 7.

7 $$\:DSC=100-\:\frac{2\left|A\cap\:B\right|}{\left|A\right|+\left|B\right|}$$

Dice coefficient is a measure that starts from zero and goes up to one point; a score of one signifies identity of sets. Dice loss is vital in enhancing the model’s outcomes when defining the margins of liver tumors.

##### Hausdorff loss (4%)

Hausdorff quantifies the distance between the actual and the predicted masks, where the difference is maximum at boundary locations. It is given by Eq. 8 where H(A, B) define the spatial function.

8 $$\:H\left(A,B\right)=\text{m}\text{a}\text{x}(h\left(A,B\right),\:h\left(B,A\right))$$

h given by Eq. 9

9 $$\:h={{max}}_{a\in\:A}{min}_{b\in\:B}||a-b||$$

It eliminates the possibility that the prediction deviates far from the actual liver tumors’ boundary, improving segmentation accuracy.

This is done by incorporating the focal loss at a 27% contribution, Dice loss at a 69% contribution, and the Hausdorff loss at a minimal 4% contribution, which together make a full, rounded loss function for the segmentation performance.

This approach utilizes a combined loss function, integrating the three individual loss functions (Focal Loss, Dice Loss, and Hausdorff Loss) with their respective weights to create a comprehensive segmentation loss. The function is defined as:

10 $$\:Loss=0.27*FL+0.69*DL+0.04*HD\:$$

Equation 10 Showcases the combined loss function helps optimize the performance of the segmentation model by balancing the benefits of each loss component, where Focal Loss focuses on handling class imbalance, Dice Loss emphasizes overlap and boundary precision, and Hausdorff Loss ensures geometric accuracy of segmentation boundaries.

#### Light GBM classifier

Here, we incorporate the Light GBM classifier and the Gravitational Optimization (GO) approach to create a systematic workflow suitable for medical imaging tasks, especially for liver tumor segmentation. This process starts with data pre-processing, for which the set is carefully partitioned into training, validation, and test sets. In this input, standard pre-processing measures, including normalization and augmentation, are used to make the model less sensitive to variations in the data. Thereby, the adept performance of this stage is vital for the following model training and the outcome of the model. The basic model structure is inherited from the U-Net architecture, to which Inception modules have been added since they help to capture multi-scale features relevant for accurate segmentation. This architectural choice enables the model to capture images at different resolutions, enhancing the model’s ability to segment the tumors appropriately. The GO algorithm is then used to find the optimum weights in the integrated loss function for the deep Bayesian network. This algorithm models the force of attraction between masses: this force directs the optimization process to the parameters’ correct settings and minimizes the aforementioned loss function.

The light GBM assumption is prominent in this integrated framework. Once the liver tumors have been segmented by the U-Net + Inception model, the segmentation process’s feature maps are post-processed to fit the Light GBM classifier input. These might include mean intensity, area, their geometric measures, and quantified attributes of textural features of masks derived from the segmented tumors. The combined loss function works with the Light GBM classifier integrated with the GO algorithm, building a highly efficient approach to liver tumor segmentation. This learning from multiple loss perspectives simultaneously solves issues like class imbalance and precision of boundary detection, and altogether increases the segmentation performance. Finally, this framework can enhance tumor detection accuracy in the clinical environment, leading to better diagnosis results and improving the quality of patients’ lives.

#### Hyperparameters

This Table 4 Hyperparameters for Gravitational Optimisation presents the hyperparameters essential for training the model. Integrating these parameters, particularly the weights assigned to each loss function, allows for precise tuning, enhancing the model’s performance in liver tumor segmentation. By optimizing these values through the Gravitational Optimization algorithm, the objective is to achieve a high level of accuracy and robustness in identifying liver tumors within medical imaging data.

**Table 4:** Hyperparameters for gravitational Optimization.

Hyperparameter	Value
Learning Rate (Segmentation)	0.001
Batch Size	32
Number of Epochs	100
Focal Loss Weight	0.27
Dice Loss Weight	0.69
Hausdorff Loss Weight	0.04
Inception Modules	3
Optimizer	Adam
LightGBM Learning Rate	0.05
LightGBM Max Depth	6
LightGBM Number of Leaves	40
LightGBM Feature Fraction	0.9
LightGBM Bagging Fraction	0.8

### Unet + Inception (UI)

The Multi-Level Inception ResNet with Bottleneck Aggregation for UIGO algorithm is designed to enhance deep feature extraction and aggregation in a segmentation framework. It builds upon a hierarchical structure combining Inception-ResNet blocks for multi-scale convolutional representation and bottleneck layers to reduce computational cost without losing essential information as shown in Algorithm 2.

- **Feature Extraction (Step 1)**:

The input image is passed through a series of Inception-ResNet blocks combining varied receptive fields (e.g., 1 × 1, 3 × 3, 5 × 5) with residual learning, ensuring rich spatial encoding and gradient stability. After each block, a bottleneck layer compresses the feature maps, reducing the number of channels to improve efficiency.

- **Hierarchical Aggregation (Step 2)**:

Bottleneck outputs are hierarchically connected using skip pathways. Higher-level feature maps are upsampled and added to lower-level ones, enabling deep skip fusion across levels. This promotes better localization and semantic context fusion — a key need in medical image segmentation.

- **Final Fusion and Output (Step 3)**:

All aggregated features are concatenated and passed through convolutional layers to produce a refined feature map. This map captures global context and local structure, achieving high segmentation accuracy.

**Algorithm 2:**
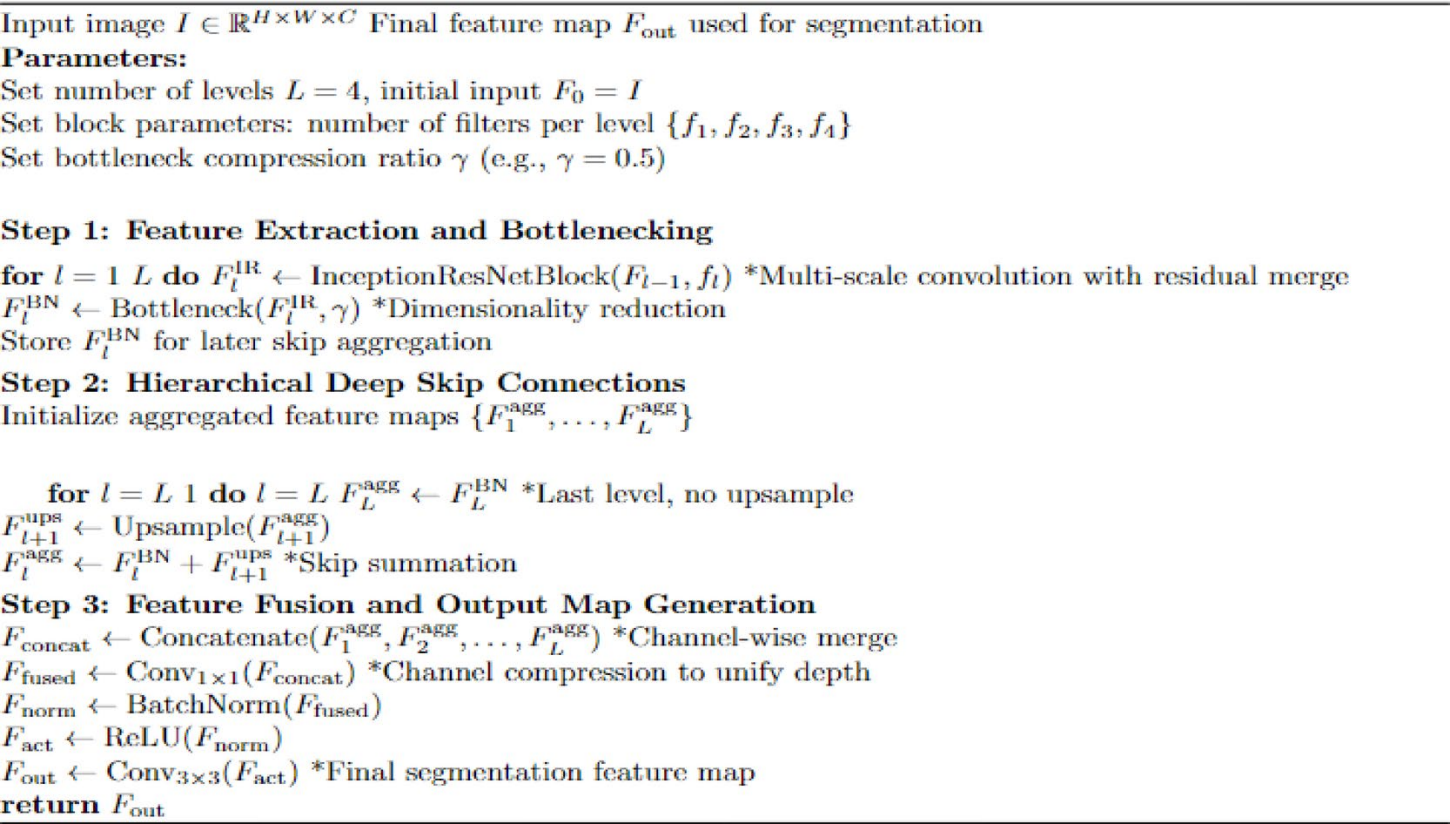
Multi-level inception ResNet with Bottleneck Aggregation for UIGO.

### Encoder

**Fig. 7:**
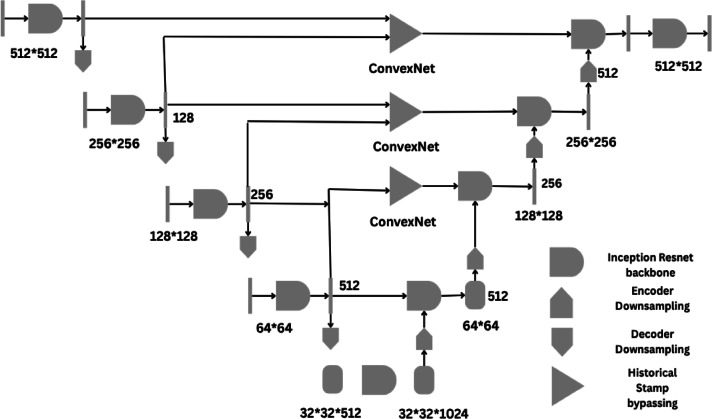
Overall Architecture of the Proposed UIGO for Liver and Tumor Segmentation.

The encoder in a U-Net + Inception architecture, as shown in Fig. 7, is mainly responsible for feature extraction in the context of input images. In this case, the encoder consists of several convolutional layers that gradually decrease input spatial dimensions and increase the number of feature maps. This work focuses on investigating the U-Net architecture for medical image segmentation while leveraging the unique architecture of U-Net, which consists of skip connections to preserve spatial information.

A U-Net architecture generally uses several convolutional blocks for the encoder block, with down-sampling layers in between. Several convolutional filters are used in each convolutional block to allow the network to learn different abstractions. The pooling layers, usually, involve max pooling; they ease the reduction of the number of feature maps so that the model can attend to the global framework at the cost of local variation. Integrating the Inception modules into the encoder increases its ability to extract multiscale information. The Inception module combines parallel convolutional branches with different kernel sizes, facilitating learning spatial hierarchies. This architecture solves the heterogeneity of the sizes of the objects in the medical images since tumors come in various sizes and shapes. Through these convolutional operations, a richer view of the data features is constructed, consequently achieving better performance in tumor segmentation-related problems. The encoder also positively determines the efficiency of the segmentation process. Besides, by employing a bottleneck architecture, which compresses the dimensionality of the feature maps before passing them to the next layer, the encoder can be optimized at a given measure of accuracy. This architecture allows the model to process large medical image datasets while producing high-quality segmentation results required for clinical applications. In sum, the function of the encoder in the proposed U-Net + Inception is to establish a solid and effective basic feature extraction and form a result for decoding and segmentation.

The proposed UIGO framework employs a multi-scale encoder-decoder architecture that integrates Inception-ResNet backbone modules for rich hierarchical feature extraction and historical stamp bypassing at each resolution level to preserve spatial and contextual information across layers. The input image of size 512 × 512 undergoes progressive down-sampling through encoder blocks at four levels: 256 × 256, 128 × 128, 64 × 64, and 32 × 32. Each level encodes local-global feature representations using Inception-ResNet modules. These features are concatenated at the bottleneck (32 × 32 × 1024) before decoding in a symmetric upsampling path. The decoder path also receives skip connections from the corresponding encoder stages and the historic stamp modules to restore fine-grained details. The final output is reconstructed back to the original resolution (512 × 512) via convolution and upsampling layers. The legend on the bottom right describes the modular components used across the pipeline. This design enhances segmentation accuracy and boundary delineation while ensuring spatial consistency through multilevel feature fusion.

### Decoder

The decoder in a U-Net + Inception architecture helps reshape the output segmentation mask from the encoded feature Representations. This section is intended to reconstructively interleave the reduced feature maps emerged from the encoder with the original input spatial resolution. The decoder also uses transposed convolution, or what some refer to as deconvolution, or a set of up-sampling layers with a convolution operation. The decoder in the U-Net architecture is described by its similarity to the encoder in its structure. This includes a set of operations, Up sample, which increase the spatial dimensions of feature maps at each layer by a factor of two. Skip connections with corresponding encoder layers accompany the up-sampling; thus, the decoder benefits from downscaled high-res features. These skip connections enable some spatial details important in distinguishing tumor boundaries in medical images. Extending the Inception modules into the decoder overcomes these limitations and provides better training of the segmentation output. Compared to the encoder, Inception modules make it possible for the decoder to capture and decode the multi-scale representation, so that the decoder can decode the fine-grained features while simultaneously considering the big picture view. This capability is especially beneficial in identifying different segments of the liver tumor since they may have irregular shapes.

Furthermore, the decoder should consider more considerations; that is, the computational complexity in achieving good segmentations should be optimized. Using a chain of bottleneck structures and a prudent choice of the number of feature maps on different layers, the decoder makes it possible to solve the problem of the trade-off between increasing the model’s complexity and its efficiency. Finally, the decoder of U-Net + Inception significantly contributes to obtaining high-quality segmentation masks and the corresponding refining for achieving comparable clinical performances.

### Bottleneck

In the U-Net + Inception model, the bottleneck means the maximum dimensionality of feature representation at the middle of the encoder and the decoder. This stage is critical as it helps focus on the input data’s most important aspects, excluding the unimportant ones from the process. Indeed, the bottleneck structure increases the model’s effectiveness in performing various tasks, such as liver tumor segmentation. When considering the U-Net + Inception framework, the bottleneck is converting into fewer feature maps using the Inception modules while retaining essential details. By using 1 × 1 convolutions within the Inception modules, the model can gain rather heavy compression, but along with that, it does not lose crucial information. Apart from helping address the problem of resource usage in a model, dimensionality reduction can also enhance the prospects of generalization to unseen data.

The bottleneck helps the feature learned at different levels of abstraction into the encoder. The bottleneck design also introduces the complexity of choosing between the levels of complexity and performance. In this context, one can identify two directions in the architecture’s design: increasing the number of Inception modules or increasing the number of feature maps per module, such that the bottleneck remains appropriately restrictive while preserving the model’s representational capabilities. Finally, the bottleneck part in the proposed network, namely U-Net + Inception, is the key factor in improving the segmentation performance for liver tumors.

### Inception backbone

In this architecture, the backbone of U-Net, thus Inception, is used for feature extraction, which affects the degree of segmentation. In this context, the backbone includes the fundamental network upon which the first features are extracted before the additional step in the U-Net structure. In most cases, some common CNN architectures, including VGG, ResNet, or Inception, can be used to extract feature weights, which have proven to be proficient in identifying different image patterns.

**Fig. 8:**
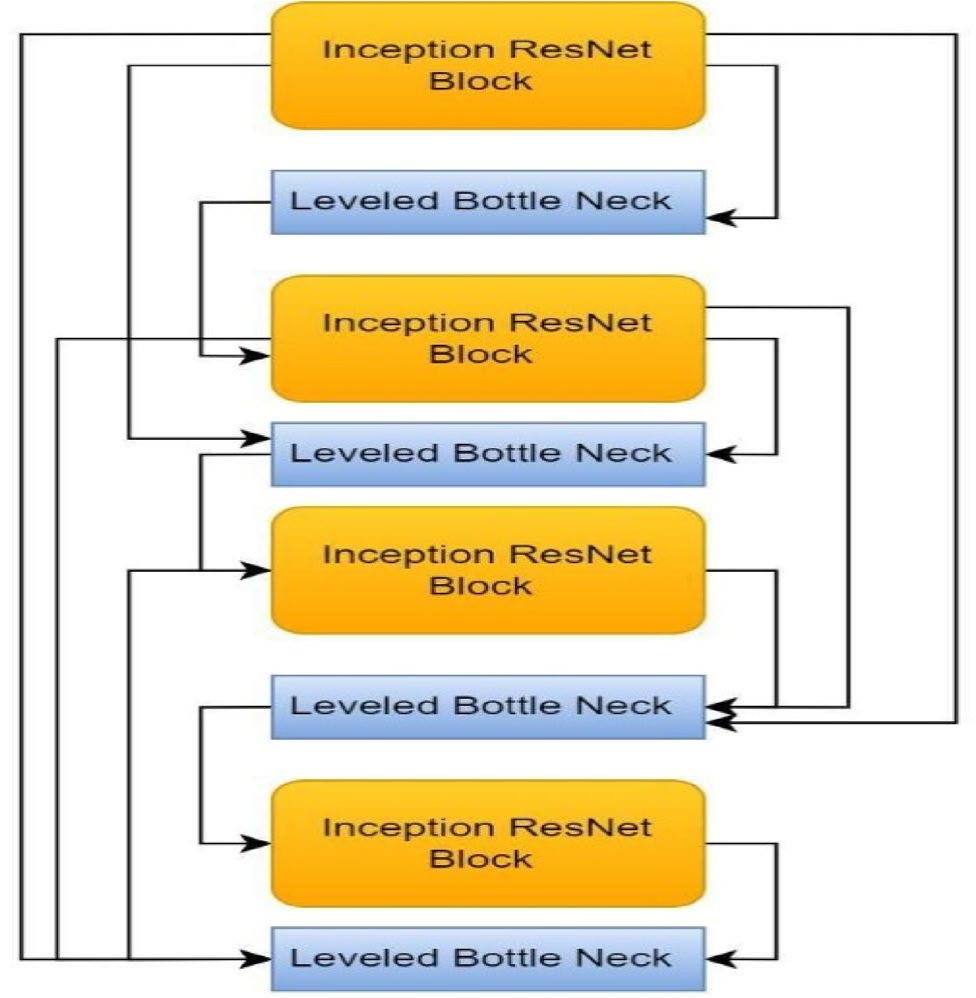
Inception backbone.

Depending on the choice of the backbone, the model can be easily generalized to different imaging modalities, which assumes increased importance in medical applications such as liver tumor segmentation. Due to the presented design, the Inception architecture is especially valuable as the backbone, as it includes many parallel convolutional paths with different kernel dimensions. This allows the network to learn multiple feature representations in parallel, reconstructing high variance in structure size and location more effectively. However, the backbone is required to minimize the total computational cost of the model as well. With the transfer learning, weights are obtained from the large dataset, then the U-Net + Inception architecture (Fig. 8 Inception backbone) has an overall higher convergence rate and better performance with fewer epochs. This is particularly important in medical imaging, where the annotated datasets are often scarce.

Unfortunately, most U-net-based models do not incorporate a rich hierarchical representation of features into the architecture inherent to Inception modules, since the model needs to understand the complex dependency of various regions in the image. This capability is instrumental, especially in delineating different types of liver tumors because these lesions are often morphologically somewhat similar. Finally, the proposed U-Net + Inception has a reference structure based on the backbone that provides the model with high performance and versatility for its application in clinical practice. Integration of Inception modules into the structure of the U-Net successfully solves several issues that may be present in medical image segmentation, both in general and specifically for liver tumors. Identifying the multi-scale feature is one of the main reasons for using Inception. Tumors can be of different sizes and forms; therefore, it is inconceivable that a single kernel can accommodate all these changes. The Inception module is proposed to have parallel convolutional branches, which use different kernel sizes to enable the model to simultaneously detect tumor features at various scale levels, improving the detection of tumors across different imaging conditions.

Further, dimensionality reduction is added in that the Inception architecture utilizes 1 × 1 convolutions to reduce the number of parameter sets, but to preserve the necessary information. This characteristic is particularly beneficial in medical imaging, where the computation is occasionally low. Through the model’s simplicity, Inception decreases the number of computations required to identify image patterns without jeopardizing the model’s efficiency, as is essential for real-time operation in clinics. Inception modules facilitate better feature fusion by allowing information from different convolutional branches to join to enhance the abstract feature representation capability. This fusion mechanism improves the description of complex features within liver tumors, which will ultimately produce better results for the segmentation of the structure. Moreover, due to the structure of Inception, the hierarchical preview of features is facilitated, thus enabling the formation of an enhanced flow of information in the model when coming up with the results.

## Experiments and analysis

The training of the proposed model was executed in two distinct phases: segmentation and classification. Initially, the segmentation sub-network—based on an enhanced U-Net structure integrated with an Inception-ResNet backbone and levelled bottleneck units—was trained independently to delineate liver and tumor regions accurately. The pre-processed input CT/MRI slices, standardized to a spatial resolution of 512 × 512 pixels, were normalized using min–max scaling to a [0,1] intensity range. The 3D volume data from LiTS, CHAOS, and 3D-IRCADb1 datasets (in Table 5) were resampled to a uniform voxel spacing of 1 × 1 × 1 mm to maintain anatomical consistency across patients and scanners.

A comprehensive data augmentation pipeline was employed using the Albumentations library to enhance the model’s generalizability and prevent overfitting. Augmentations were applied on-the-fly with the following transformations: random rotations within ± 15°, horizontal and vertical flips, elastic deformations, brightness/contrast adjustments, and adaptive histogram equalization using CLAHE. Additionally, light Gaussian noise was added with a standard deviation range of 0.01–0.05 to simulate scanner variability. Each augmentation had a trigger probability ranging from 0.2 to 0.5.

For segmentation training, the Adam optimizer was used with an initial learning rate of 1e-4, and the model was trained for 150 epochs with early stopping enabled (patience = 15 epochs) based on the validation Dice score. The composite loss function used was a weighted sum of Dice Loss and Binary Cross-Entropy (BCE) to balance overlap accuracy and pixel-wise classification. Learning rate decay was dynamically controlled via the ReduceLROnPlateau scheduler.

Upon obtaining accurate segmentation masks, the second phase of training was focused on classification. Features were extracted from segmented regions using deep latent representations and passed through a Gravitational Optimization (GO) module. The GO algorithm was initialized with a particle population of 25, iterated over 100 generations, and optimized based on classification consistency and segmentation-aware salience. The best feature subset obtained through GO was then used to train a LightGBM classifier, which offered robust gradient-based boosting with superior handling of structured data and imbalance.

All experiments were conducted on a high-performance system equipped with a NVIDIA RTX A6000 GPU (48 GB VRAM), Intel Xeon Gold 6226R CPU @ 2.90 GHz, and 256 GB of RAM. The implementation was done using PyTorch v2.0 with CUDA 11.8, and training progress, validation metrics, and visualizations were tracked using Weights & Biases and TensorBoard for reproducibility and monitoring.

**Table 5:** Dataset summary and Splits.

Dataset Name	Class	Training Instances	Validation Instances	Testing Instances	Total Instances	Number of Patients
LiTS	Liver	141	40	20	201	201
LiTS	Tumor	141	40	20	201	201
3D-IRCADb1	Liver	1860	532	266	2658	20
3D-IRCADb1	Tumor	1860	532	266	2658	20
3D-IRCADb1	Liver	1531	437	219	2187	20
3D-IRCADb1	Tumor	1531	437	219	2187	20
CHAOS	Liver	4485	1281	641	6407	40
CHAOS	Tumor	4485	1281	641	6407	40

### Metrics

The study utilizes a detailed evaluation plan for the proposed hybridized model. The study uses accuracy (A), precision (P), recall (R), F1 score (F1S), and specificity (SP) as per the Table 6 Metric Formulas. It uses True Positive or T+, False Positive or F+, True Negative or T-, and False Negative or F-.

**Table 6:** Metric Formulas.

Metric	Formula
A	$\:\frac{(\text{T}-)+(\text{T}+)}{\left(\text{T}-\right)+\left(\text{T}+\right)+\left(\text{F}+\right)+(\text{F}-)}$
P	$\:\frac{\left(\text{T}+\right)}{\left(\text{T}+\right)+\left(\text{F}+\right)}$
R	$\:\frac{\left(\text{T}+\right)}{\left(\text{T}+\right)+\left(\text{F}-\right)}$
F1S	$\:\frac{2\text{*}\text{P}\text{*}\text{R}}{\text{P}+\text{R}}$
SP	$\:\frac{\left(\text{T}-\right)}{\left(\text{T}-\right)+\left(\text{F}+\right)}$
Sensitivity	$\:\frac{\left(\text{T}+\right)}{\left(\text{T}+\right)+\left(\text{F}-\right)}$

The study employs a comprehensive evaluation plan to assess the performance of the proposed hybridized model for liver tumor segmentation, utilizing several key metrics: accuracy (A), precision (P), recall (R), F1 score (F1S), and specificity (SP). Accuracy measures the overall correctness of the model, reflecting the ratio of correctly predicted instances (both true positives and true negatives) to the total cases, thereby indicating the model’s effectiveness in correctly identifying liver tumors. Precision evaluates the proportion of accurate optimistic predictions against the total optimistic predictions (true positives plus false positives), highlighting the model’s ability to avoid false alarms—an essential consideration in clinical settings where misdiagnosis can lead to unnecessary interventions. Recall, or sensitivity, measures the proportion of true positives identified out of all actual positives (true positives plus false negatives), underscoring the model’s capability to detect as many tumors as possible and minimize the risk of overlooking malignancies. The F1 score, as the harmonic mean of precision and recall, provides a single metric that balances the trade-off between them, making it particularly useful when dealing with imbalanced datasets where negative cases vastly outnumber positive ones. Finally, specificity assesses the proportion of true negatives correctly identified among all actual negatives (true negatives plus false positives), indicating how well the model recognizes non-tumor areas and thus reduces unnecessary interventions. Together, these metrics, grounded in fundamental classification concepts—True Positive (T+), False Positive (F+), True Negative (T-), and False Negative (F-)—form a robust framework for evaluating the efficacy of the proposed model in liver tumor segmentation tasks.

### Ablation study

We conducted a series of ablation experiments to thoroughly evaluate the architectural and functional design choices of the proposed UIGO model. These experiments isolate key components such as backbone modules, generative augmentative strategies, and loss function configurations to quantify their individual and combined contributions to the overall performance. In particular, we examined how different feature extractors and preprocessing modules impact segmentation robustness. Furthermore, in subsection 5.2.1, we present a comparative analysis of various loss function strategies. We demonstrate how our composite loss formulation balances region accuracy, boundary sensitivity, and class imbalance—key factors in medical image segmentation.

Table 7 Presents an ablation analysis exploring the incremental contributions of each component of the UIGO pipeline, ranging from preprocessing strategies to architectural enhancements. The baseline model (UIGO-A1) lacks preprocessing and augmentation and uses a standard FCN decoder with an Inception backbone. It achieves a Dice score of 0.9573 and a Hausdorff Distance (HD) of 0.0625. Introducing bilateral filtering and Gaussian blurring (UIGO-A2) as a preprocessing stage result in noticeable improvements. Bilateral filtering preserves important anatomical boundaries while reducing fine-grained noise, and Gaussian blurring smoothens texture inconsistencies, which lead to better edge learning and improve downstream model convergence. This preprocessing alone leads to a ~ 0.45% Dice gain.

Further enhancements come from using GAN-based augmentations. While UIGO-A2 employs vanilla GANs, UIGO-A3 uses CycleGAN, which offers domain-adaptive transformations (e.g., MRI to CT), leading to a better Dice score (0.9642) and reduced HD (0.0536). Switching to more profound and expressive backbones yields the UIGO-B1 and B2 models. ResNet-50 shows slight gains over Inception, but NASNet (UIGO-B2) performs better due to its learned architecture search capabilities. Building upon this, UIGO-C1 integrates a Residual FCN, further deepening the network and boosting performance. The final configuration, UIGO-C2, combines all optimal elements: preprocessing, CycleGAN augmentation, NASNet backbone, and a Cross RCN-LSTM-based FCN decoder. This model achieves the best performance across all metrics (Dice: 0.9690 ± 0.0020, HD: 0.0476 ± 0.0014), confirming the cumulative value of each design choice. These improvements are significant in clinical segmentation, where anatomical clarity and consistency are critical.

**Table 7:** Ablation study of UIGO Variants – Preprocessing, augmentation, backbone, and FCN Architecture.

Model Variant	Preprocessing	Augmentation	Backbone	FCN Type	Dice Score	Hausdorff Distance
UIGO-A1	None	None	Inception	Standard FCN	0.9573 ± 0.0041	0.0625 ± 0.0032
UIGO-A2	Bilateral + Gaussian Filtering	GAN	Inception	Standard FCN	0.9619 ± 0.0036	0.0564 ± 0.0029
UIGO-A3	Bilateral + Gaussian Filtering	CycleGAN	Inception	Standard FCN	0.9642 ± 0.0032	0.0536 ± 0.0025
UIGO-B1	Bilateral + Gaussian Filtering	CycleGAN	ResNet-50	Standard FCN	0.9611 ± 0.0038	0.0552 ± 0.0026
UIGO-B2	Bilateral + Gaussian Filtering	CycleGAN	NASNet	Standard FCN	0.9629 ± 0.0035	0.0521 ± 0.0023
UIGO-C1	Bilateral + Gaussian Filtering	CycleGAN	NASNet	Residual FCN	0.9661 ± 0.0029	0.0502 ± 0.0019
UIGO-C2 (Final)	Bilateral + Gaussian Filtering	CycleGAN	NASNet	Cross RCN–LSTM FCN	**0.9690 ± 0.0020**	**0.0476 ± 0.0014**

#### Loss function: comparative study

An ablation study was conducted using several loss configurations to evaluate the influence of different loss function strategies on the performance of the UIGO model. As shown in Table 8 Using Dice Loss alone resulted in a Dice score of 0.9482 ± 0.006, which, while reasonable, was accompanied by a relatively high Hausdorff Distance (0.0721 ± 0.0043). This suggests that although Dice Loss effectively captures region overlap, it lacks sensitivity to boundary details, which is critical in segmenting irregular tumor shapes.

Introducing Hausdorff Loss alongside Dice led to noticeable improvements in Dice score (0.9567 ± 0.004) and boundary delineation (Hausdorff Distance reduced to 0.0613 ± 0.0031). This indicates that incorporating geometric error helps the model better approximate tumor boundaries. However, without explicitly addressing class imbalance, the model’s ability to accurately predict smaller or sparse tumor regions remains suboptimal.

To tackle this, the Dice + Focal Loss configuration was explored, which improved both Dice (0.9591 ± 0.005) and Hausdorff Distance (0.0570 ± 0.0027) compared to earlier combinations. By down-weighting easy examples and focusing training on more complicated cases, Focal Loss provided better balance across tumor and background regions.

The proposed tri-composite loss—combining Dice, Focal, and Hausdorff Loss—achieved the best overall performance, with a Dice score of 0.9690 ± 0.0020, IoU of 0.9509 ± 0.002, and the lowest Hausdorff Distance of 0.0476 ± 0.0014. This combination capitalized on region-level accuracy, class imbalance mitigation, and boundary precision, offering a robust and holistic loss design.

Furthermore, experiments with Tversky Loss (which generalizes Dice by incorporating penalties for false positives and negatives) and Focal Tversky Loss showed promising results, especially under imbalance. The Focal Tversky configuration achieved a Dice of 0.9610 ± 0.004 and a Hausdorff Distance of 0.0544 ± 0.0021, underscoring its potential. However, the proposed loss outperformed all alternatives, suggesting that its balanced design effectively guides the model toward more precise and stable convergence across complex tumor scenarios.

**Table 8:** Ablation study of loss function combinations on liver tumor Segmentation.

Loss Function(s) Used	Dice Score (↑)	IoU Score (↑)	Hausdorff Distance (↓)	AUC (↑)
Dice Loss Only	0.9482 ± 0.006	0.9205 ± 0.007	0.0721 ± 0.0043	0.9931
Dice + Hausdorff	0.9567 ± 0.004	0.9338 ± 0.005	0.0613 ± 0.0031	0.9942
Dice + Focal	0.9591 ± 0.005	0.9372 ± 0.004	0.0570 ± 0.0027	0.9951
Dice + Focal + Hausdorff (Proposed)	**0.9690 ± 0.002**	**0.9509 ± 0.002**	**0.0476 ± 0.0014**	**0.9978**
Tversky Loss (α = 0.7, β = 0.3)	0.9578 ± 0.005	0.9349 ± 0.006	0.0602 ± 0.0032	0.9956
Focal Tversky Loss (α = 0.7, β = 0.3, γ = 0.75)	0.9610 ± 0.004	0.9402 ± 0.004	0.0544 ± 0.0021	0.9964

### Qualitative analysis across datasets

#### 3D-IRCADb1

**Fig. 9:**
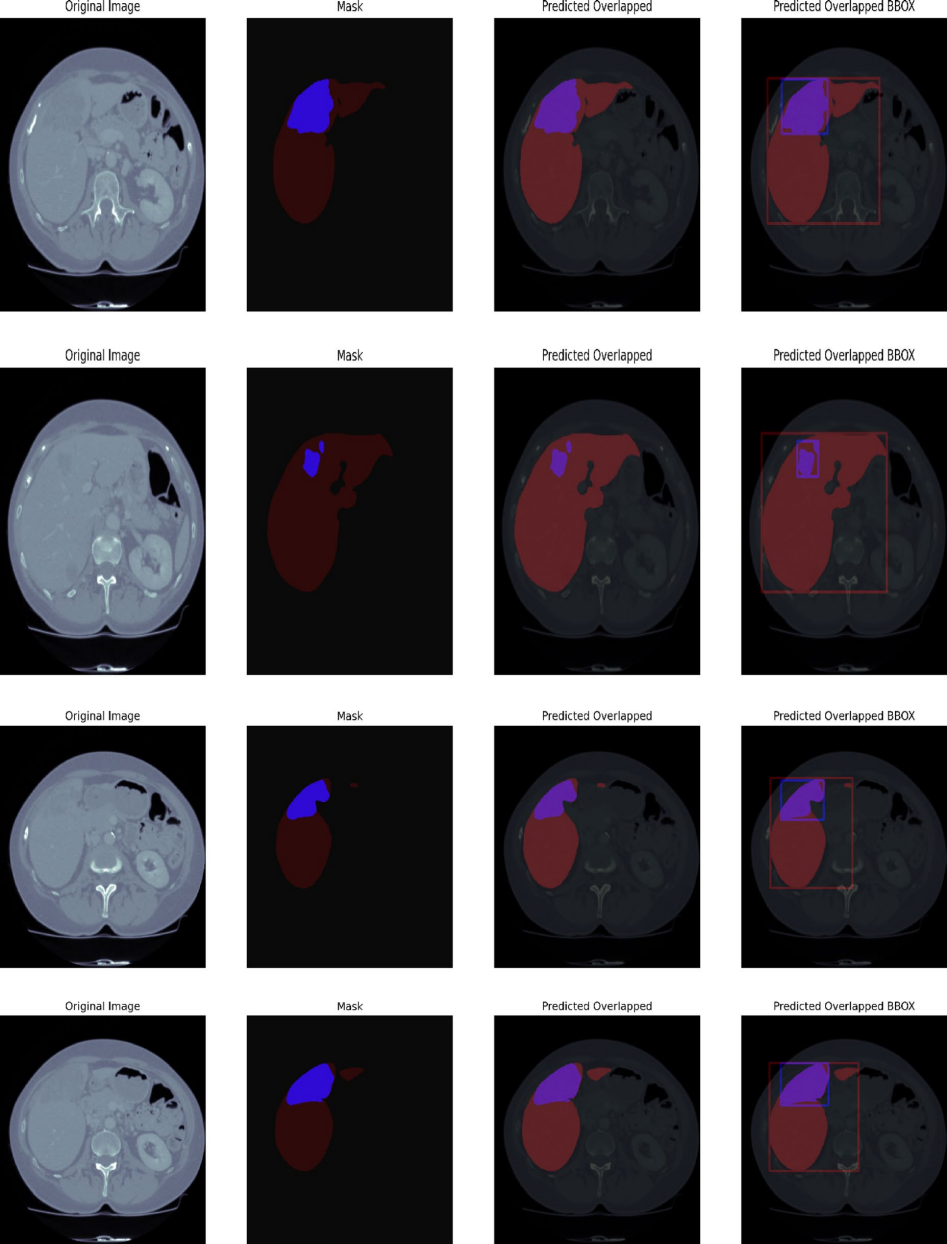

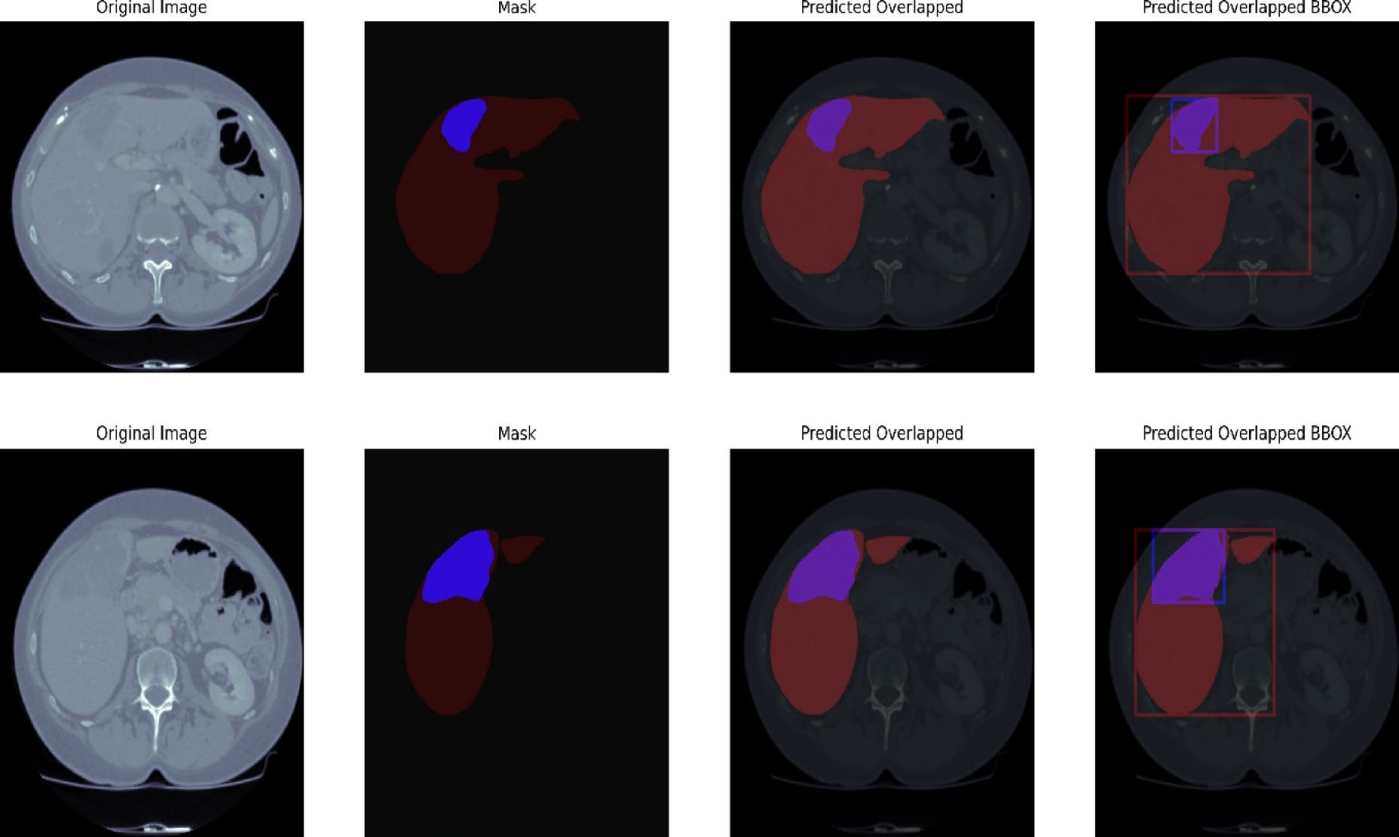
3D-IRCADb1 Qualitative Visualization of Liver and Tumor from UIGO architecture with overlap and boundary box.

This Fig. 9 3D-IRCADb1 Qualitative Visualization of Liver and Tumor from UIGO architecture with overlap and boundary box summarizes a qualitative comparison of the liver and tumor segmentation of the 3D-IRCADb1 data set using the UIGO structure, which is a U-net with Inception and Gravitational Optimization. This visualization is aimed to support the evaluation of our proposed model to localize the liver and tumor regions in medical images, and its potential in the improvement of clinical insights.

The predicted overlap is highlighted dominantly in the visualization to reveal such regions where the UIGO model is accurate in projecting the liver and the tumor. This is the bitmap where there is an overlap between the predicted tumor segmentation and the ground truth liver segmentation. A strong overlap means that the UIGO model has been well-trained in distinguishing boundaries between liver and tumor tissues, which is essential for an accurate diagnosis and treatment. Thus, by proceeding with such a clear definition of these areas, the given model finds its application in the enhancement of outcomes in clinical contexts, important of which is the localization of the tumor.

Further, we observe that each tumor region is surrounded by a BBox that gives an overview of the localization and size of the detected tumor in the liver. The bounding box is used to draw the clinician’s attention to the size and position of the tumor within the given image. Besides improving the interpretability of the segmentation results, this feature enables quantitative evaluations of the tumor width. Such information is useful in assessing the tumor status or even how it responds to therapy through the model prediction of healthcare practitioners.

#### Liver tumor segmentation (LiTS 201)

**Fig. 10:**
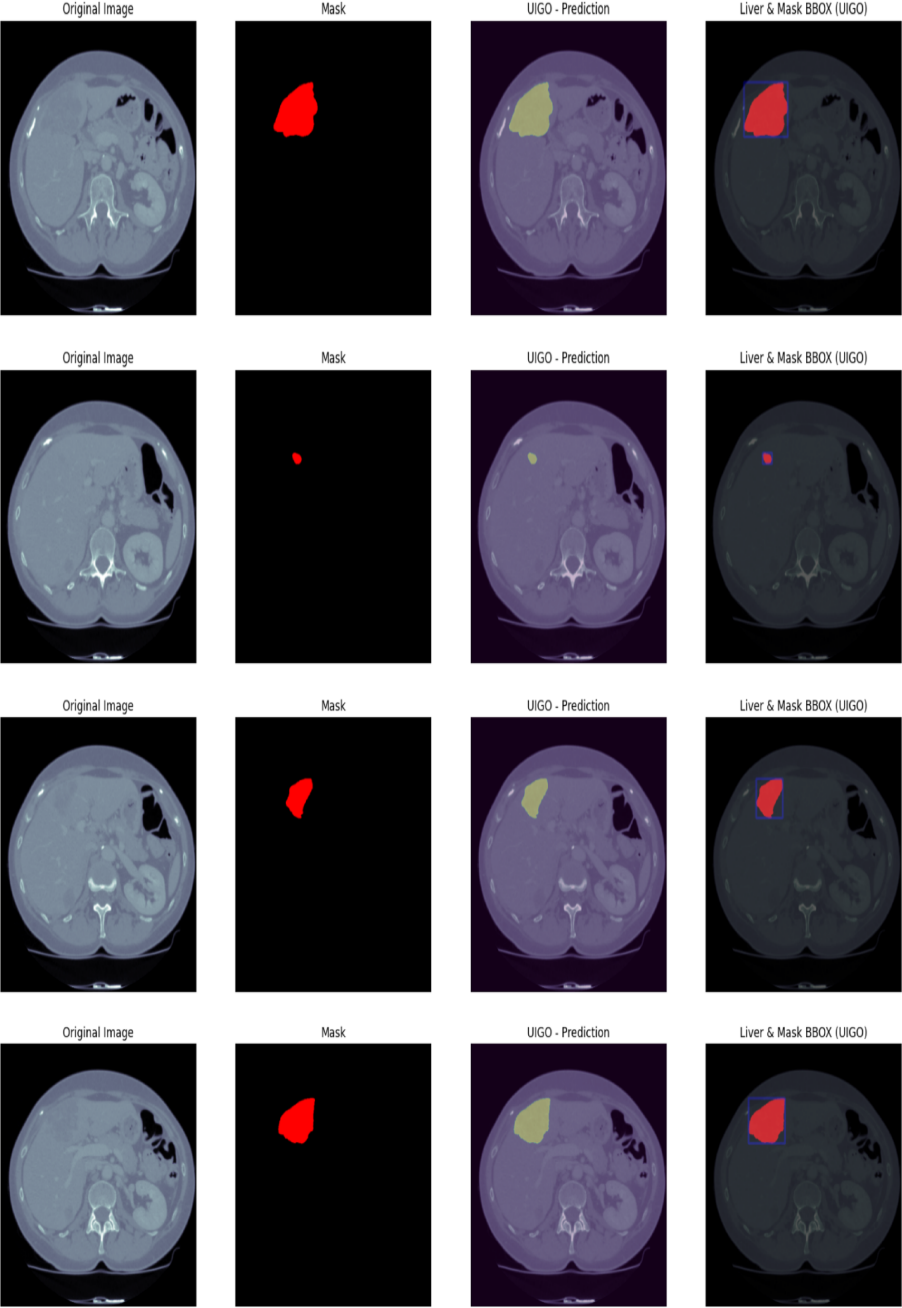

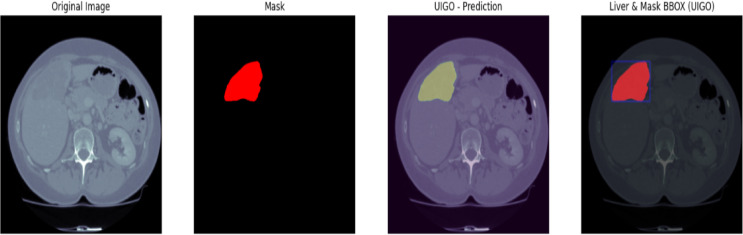
LiTS Qualitative Visualization of Liver and Tumor from UIGO architecture with overlap and boundary box.

The Fig. 10 LiTS Qualitative Visualization of Liver and Tumor from UIGO architecture with overlap and boundary box The qualitative liver and tumor segmentation results based on the proposed UIGO (U-Net with Inception and Gravitational Optimization) architecture developed on the LiTS (Liver Tumor Segmentation) dataset. Of them, the visualization of the predicted overlap between the segmented liver and tumor regions is the most valuable, as it demonstrates the ability of the proposed model to define these vital anatomical structures precisely.

This overlap area is where the model successfully identified a tumor within the liver, thereby underscoring the localized nature of the UIGO architecture. Moreover, BBox is employed to contain the discovered tumor areas so that the sizes and positions of the tumors can be easily recognized. For this reason, this qualitative analysis of the UIGO model has highlighted the model’s ability to improve the workflows of clinical settings, including the diagnostic reasoning of radiologists and oncologists in their management of cancer cases. Outcomes reveal the proposed model’s efficacy in the liver tumor segmentation task.

#### CHAOS

**Fig. 11:**
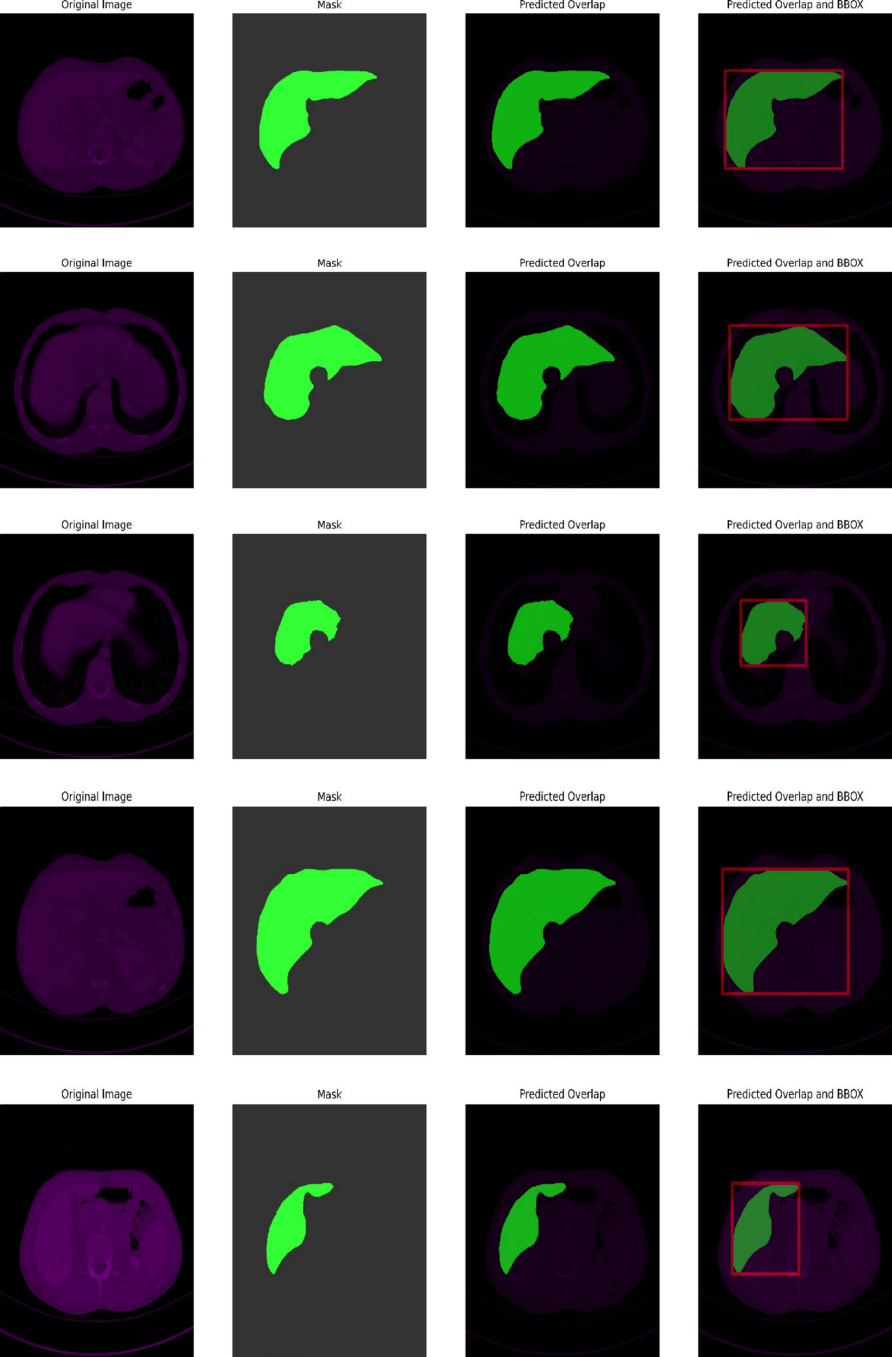

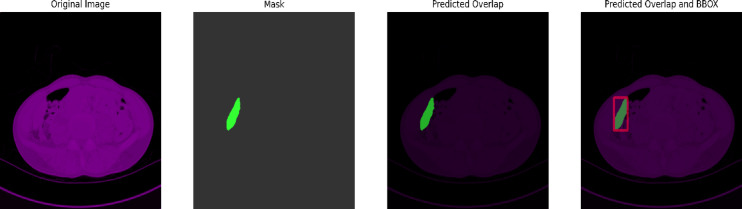
CHAOS Qualitative Visualization of Liver and Tumor from UIGO architecture with overlap and boundary box.

The percentage of segmentation accuracy for liver and tumor segmentation utilizing the CHAOS dataset on the proposed UIGO architecture is depicted in the Fig. 11. The depiction is quite helpful in establishing the expected coincidence of the segmented liver and tumor areas, demonstrating the model’s high ability to detect those vital anatomic structures in absorption anatomical scans.

The overlapping of our model successfully shows the region where it outlines tumor presence concerning the liver, which evidences its capacity to address complex structures. The final set of enhancements encompasses the bounding boxes (BBox), which also highlight the image sections containing the tumor based on the learning model, and allow for understanding specific tumor characteristics, namely its size and position. This qualitative analysis supplements the effectiveness of the UIGO model in clinical practice. However, the vital information necessary for diagnosing and treating patients is provided to help therapists and other health care professionals. The CHAOS benchmarking results indicate the practical validity and efficiency of the proposed model in liver tumor segmentation.

### Quantitative analysis across datasets

The metrics in Table 9, UIGO performance across Datasets, present an analysis of the UIGO architecture’s efficacy in segmenting liver and tumor structures across three datasets: LiTS, CHAOS, and 3DIRCAD. In LiTS, therefore, the loss for liver segmentation was − 0.90178 with an accuracy and binary accuracy of 0.999368, suggesting the model nicely separates segmented blobs from background pixels. The AUC score of 0.999878 further supports the model’s excellent discrimination ability, and specificity and sensitivity values of 0.999718 and 0.999875 prove that the model successfully classifies the true negatives and true positives. In the tumor segmentation within the same dataset, the recorded loss is 0.010162, which, though a bit higher than the previous results, is still impressive. The accuracy and binary accuracy remain at a very satisfactory 0.998771, while an AUC of 0.99989 further suggests the model’s effectiveness in identifying tumor structures.

Using the same metrics on the CHAOS dataset, the loss is bigger: 0.301428, but the accuracy and binary accuracy are still high: 0.964359, respectively; therefore, this model has a good segmentation capability despite the increased loss. For the validation of the model to classify the difference between the liver tissues and the tumor backgrounds, the AUC score values that acknowledged its performance were as follows: the value of 0.99789, while the specificity and sensitivity values were 0.96181 and 0.994977, respectively. The detected loss in the CHAOS dataset for tumor segmentation is 0.017644, with impressive accuracy and binary accuracy of 0.99965. The AUC score stays high at 0.99789, which underlines the sound classification results, which are backed up by specificities/sensitivities of 0.99987/ 0.980444, respectively, which proves the model’s reliability when it comes to tumor detection.

**Table 9:** UIGO performance across Datasets.

Dataset	Structure	Loss	Accuracy	Binary Accuracy	AUC	Specificity	Sensitivity
LiTS	Liver	-0.90178	0.999368	0.999368	0.999878	0.999718	0.999875
	Tumor	0.010162	0.998771	0.998771	0.99989	0.999546	0.994284
CHAOS	Liver	0.301428	0.964359	0.964359	0.99789	0.96181	0.994977
	Tumor	0.017644	0.99965	0.99965	0.99789	0.99987	0.980444
3DIRCAD	Liver	0.01878	0.999887	0.999887	0.998558	0.999965	0.982717
	Tumor	0.046263	0.999971	0.999971	0.997669	0.97977	0.99999

To assess the generalization capability of the proposed UIGO model, experiments were conducted on three diverse benchmark datasets: LiTS (CT-based), 3D-IRCADb1 (contrast-enhanced CT), and CHAOS (MRI-based). These datasets differ in imaging modality, anatomical variations, and acquisition protocols, posing a realistic challenge for any segmentation framework. As summarized in Table 9, UIGO achieves consistently high Dice and IoU scores across all datasets, demonstrating that its architecture, driven by inception modules and gravitational optimization, captures domain-invariant features effectively. It is important to note that the model was evaluated without explicit domain adaptation mechanisms. This strengthens the claim that UIGO is inherently generalizable. Nonetheless, performance on heterogeneous data can be further improved through techniques like unsupervised domain adaptation, style transfer-based normalization, or domain adversarial training. Future work will explore integrating such strategies into UIGO to make it even more robust for cross-center and multi-institutional clinical deployment.

#### Complementary quantitative metrics for segmentation validation

To further strengthen the performance evaluation of the UIGO model, we computed additional classification metrics, including Precision, Recall, and the Matthews Correlation Coefficient (MCC) for both liver and tumor segmentation across all three datasets: LiTS, CHAOS, and 3D-IRCADb1. As presented in Table 10, the model consistently achieves high precision and recall values, indicating both low false-positive and low false-negative rates. For instance, in the LiTS dataset, liver segmentation achieved a precision of 0.99994 and recall of 0.99988, with an MCC of 0.99981, demonstrating almost perfect classification alignment. Even in more heterogeneous datasets like CHAOS (MRI), UIGO maintains high MCC scores above 0.98, confirming its robust and reliable performance. These metrics provide a complementary view to Dice and IoU, and affirm that the model balances sensitivity and specificity well—essential for clinical deployment.

**Table 10:** Precision, recall, and MCC for liver and tumor segmentation across Datasets.

Dataset	Structure	Precision	Recall (Sensitivity)	MCC
LiTS	Liver	0.99994	0.99988	0.99981
	Tumor	0.99901	0.99428	0.99661
CHAOS	Liver	0.99582	0.99498	0.98190
	Tumor	0.99944	0.98044	0.98950
3D-IRCAD	Liver	0.99998	0.98271	0.99125
	Tumor	0.96059	0.99999	0.97901

### Cross validation and statistical testing

Table 11 Statistical Details of K-Fold Cross show the findings of the K-fold validation where the feature selection model receives high mean and most of the time high standard deviation for the 60 validation folds encompassing loss, binary accuracy, accuracy, area under the curve, specificity, and sensitivity. The average loss, subject to a slight negativity estimate of − 0.08265, accompanied by a standard deviation of 0.37586, implies some measure of variation. This is well illustrated by its low standard deviation with a value of 0.01438 and failure rate ranging from 0.93929 to 0.99922, and the mean of 0.97814 demonstrates that the model performs well in all the folds. Binary accuracy is also relatively high, ranging about 0.98093 and revealing high classification ability.

The perfect classification in most of the folds is indicated by the slightly higher mean of the AUC of approximately 1.00119, with very little standard deviation. Specificity, which is computed by the side of the model that effectively labels negative cases, also shows high reliability with a mean of 0.97554. Sensitivity – the ability to detect positive cases, stands at 0.97964 on average, proving the stability and efficiency of the model. Based on these analyses, the outcome highlights little fluctuation within the folds, high specificity and sensitivity scores that imply the ability of the model in classifying both the positive and negative cases in the dataset.

**Table 11:** Statistical details of K-Fold cross Validation.

StatS	Fold	Loss	Accuracy	Binary Accuracy	AUC	Specificity	Sensitivity
mean	5.5	-0.082652	0.978143	0.98093	1.00119	0.975542	0.979644
std	2.89652	0.375859	0.014381	0.014568	0.017047	0.016894	0.011075
min	1	-0.898899	0.939291	0.941142	0.969981	0.933568	0.95573
25%	3	0.010031	0.972184	0.973878	0.987978	0.963122	0.970532
50%	5.5	0.017924	0.981287	0.983994	1.001589	0.978932	0.979018
75%	8	0.045249	0.987645	0.992801	1.015656	0.988494	0.988786
max	10	0.301396	0.999222	0.997678	1.028615	0.999083	0.999737

**Fig. 12:**
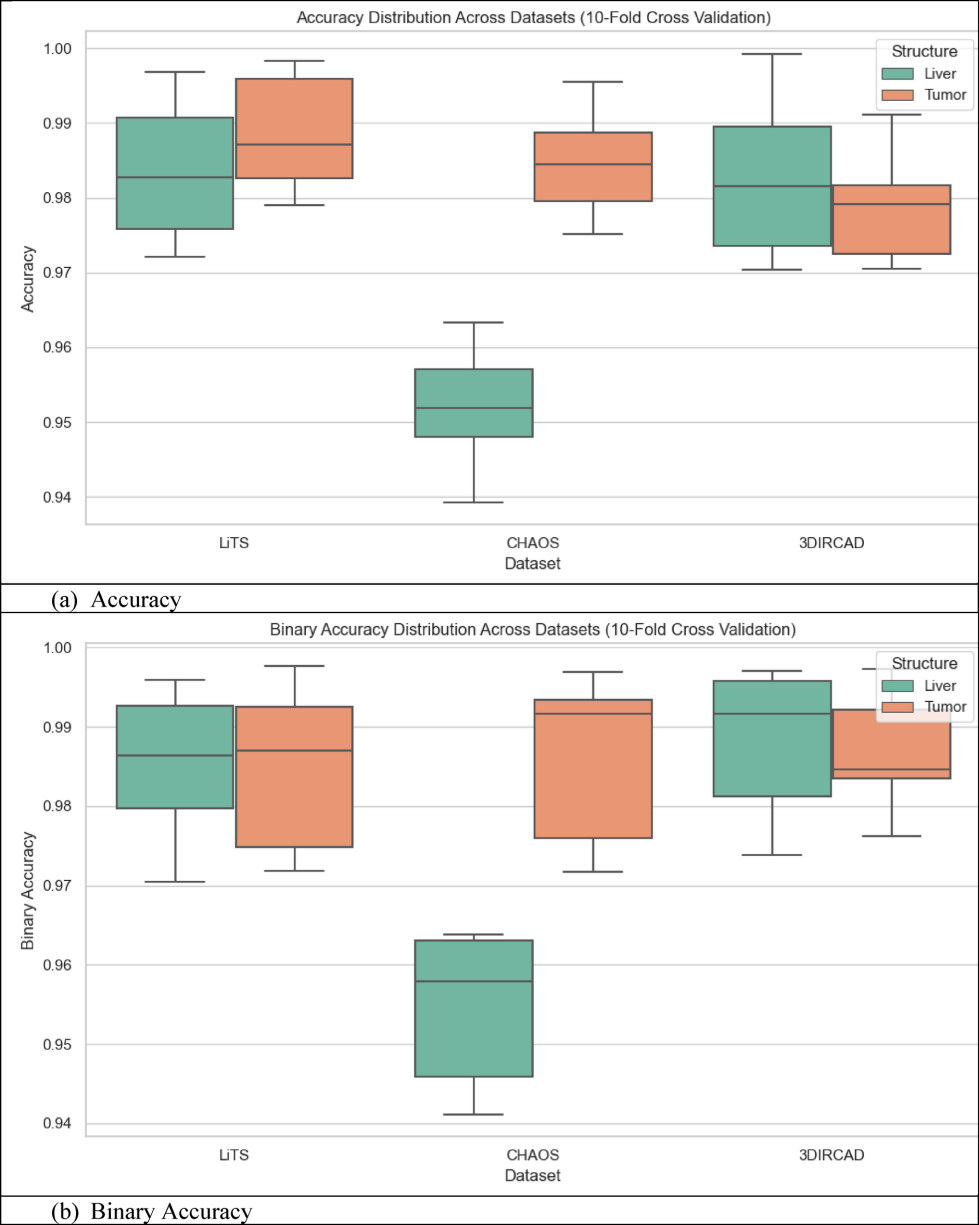

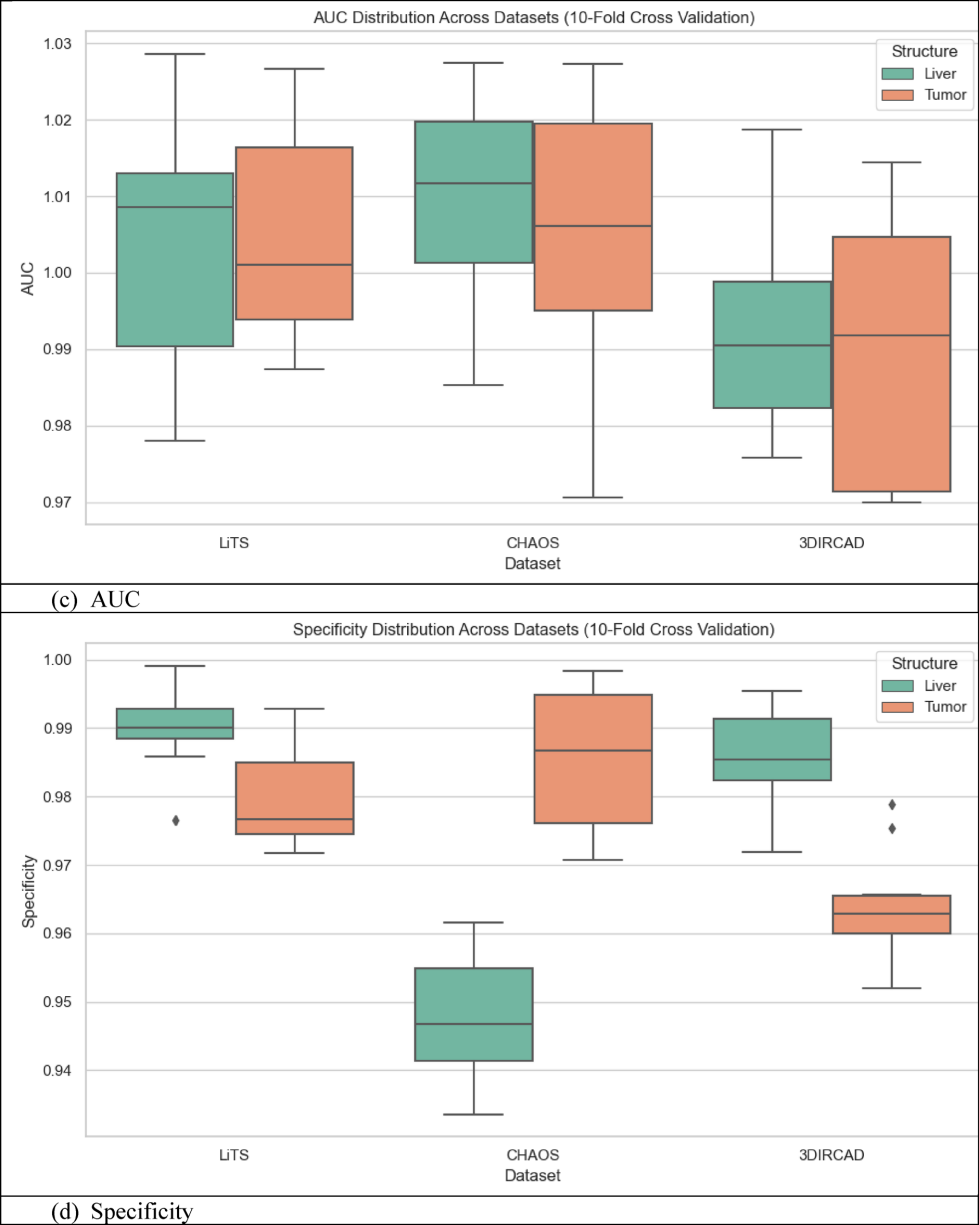

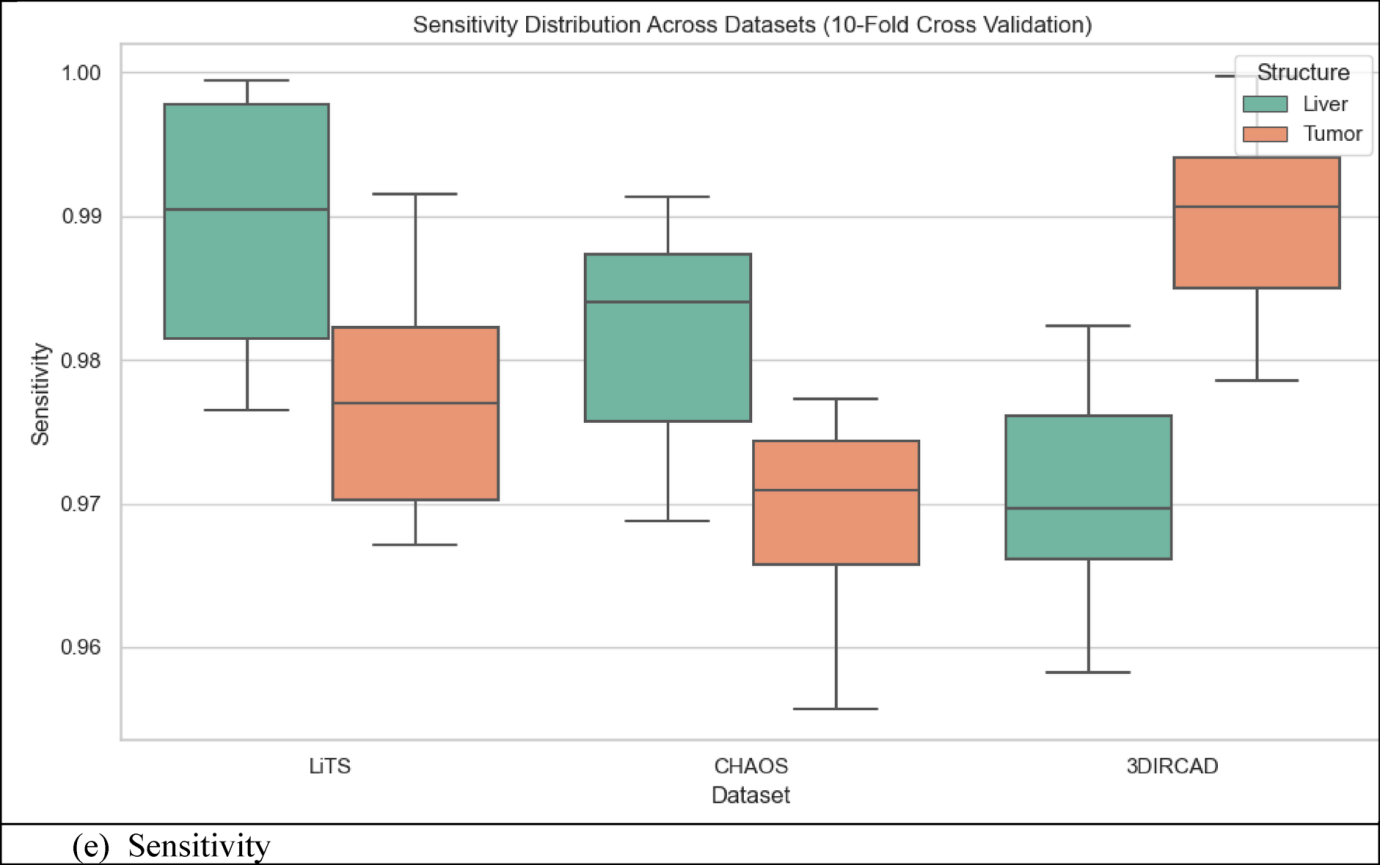
K-Fold Cross-Validation based Box Plot.

The box plots in Fig. 12, give a clear idea about the variation and exactness of the model over multiple folds. K-Fold Cross Validation extracts the dataset into K subsets or folds and then trains the model on K-1 folds while testing on another fold. The procedure is iterated K times, wherein each fold personally validates once, with all the K-cycle means used as the final performance yardstick. In the box plots, each set, that is, LiTS, CHAOS, and 3DIRCAD, has two constituents: the liver and the tumor, and the following performance indicators: loss, accuracy, binary accuracy, AUC, specificity, and sensitivity have been plotted. The plots represent the performance in 10-fold cross-validation to show the distribution of the values between the folds.

These box plots bring many insights into the model stability and generalization. Concerning the box plot, the figure range is presented in each set of data, with the observation of the interquartile range depicting the middle of the line for model performance. The former shows the level of consistency seen in folds, while the latter shows a degree of inconsistency experienced by the model. This makes understanding how well the model is likely to perform when presented with subsets of the data fairly straightforward.

#### Statistical testing

**Fig. 13:**
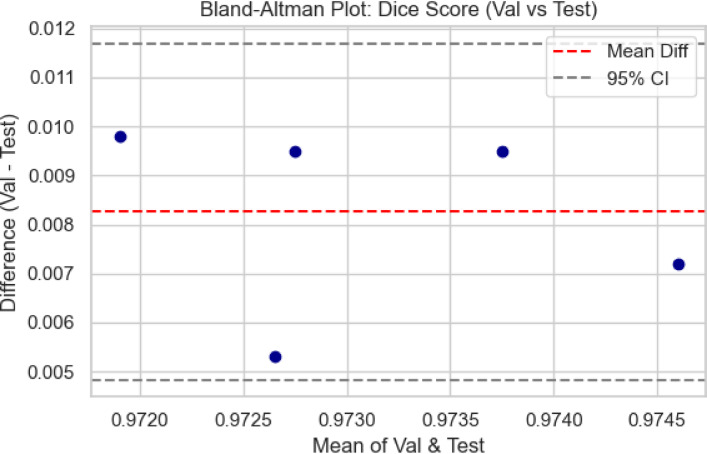
Bland-Altman Plot for the difference between validation and unseen test set.

**Fig. 14:**
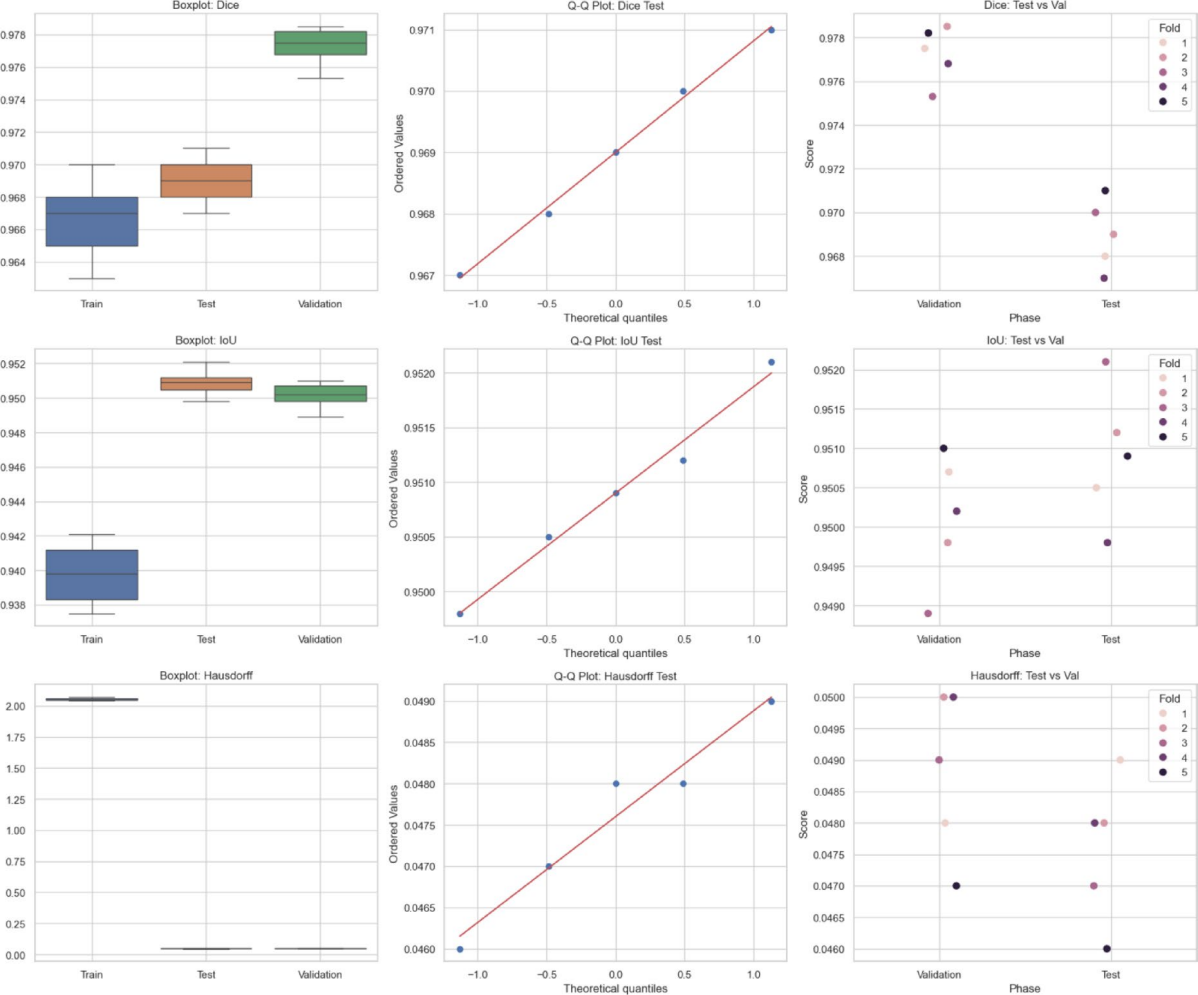
Statistical Distribution Plots across Training, Validation, and Test Sets. A set of visual analyses including boxplots, Q–Q plots, and paired strip plots for Dice, IoU, and Hausdorff Distance metrics across train, validation, and test partitions. These visualizations further confirm normality, consistent score distributions, and the generalization capability of the proposed model.

To ensure the robustness and generalizability of the proposed model and eliminate overfitting concerns, a comprehensive statistical analysis was carried out across the training, validation, and unseen test datasets. The evaluation began with formal statistical hypothesis testing, including one-way ANOVA, Wilcoxon signed-rank test, Shapiro–Wilk test for normality, and Levene’s test for homogeneity of variances. These tests were performed independently for key segmentation metrics, Dice Score, Intersection over Union (IoU), and Hausdorff Distance across the three data partitions. While ANOVA reported statistically significant differences (*p* < 0.0001) due to natural distribution shifts, the Wilcoxon test between validation and test results was non-significant (*p* > 0.05) for all metrics, indicating consistent generalization. Additionally, the Shapiro–Wilk and Levene’s test results confirmed that the data distributions were normal and variances were homogeneous, validating the assumptions required for parametric testing. The Bland–Altman method was applied to compare Dice scores from validation and unseen test sets to complement these statistical tests. As shown in Fig. 12, the mean difference between validation and test predictions was marginal (~ 0.008), and all data points fell within the 95% limits of agreement, providing strong visual evidence that the model’s performance remains stable across different subsets. The summary of test set performance is presented in Table 12, with mean Dice and IoU scores of 0.9690 and 0.9509, respectively, and extremely narrow confidence intervals. These are further supported by large Cohen’s *d* values (35.80 and 45.22), indicating high statistical significance over baseline. Table 13 Presents the full results of statistical tests across folds and data splits. Figure 13 Illustrates the Bland-Altman Plot for the Dice Score, comparing performance between the validation and unseen test sets. The differences (Val − Test) lie within the 95% confidence limits, with a mean difference of approximately 0.008, indicating strong alignment between the two sets. This supports the claim that the model generalizes well and does not significantly overfit to the validation set. In addition, Fig. 14 Showcases a series of visualizations—including boxplots, Q–Q plots, and paired strip plots—further validate distributional similarity and stability across the datasets. This multi-layered evaluation demonstrates that the model generalizes effectively and does not suffer from overfitting, affirming its reliability for application on unseen clinical data.

**Table 12:** Cross-Validation metrics on unseen test Set.

Metric	Mean	95% Confidence Interval	Cohen’s d
Dice Score	0.9690	0.9670 to 0.9710	35.80
IoU Score	0.9509	0.9498 to 0.9520	45.22
Hausdorff Distance	0.0476	0.0462 to 0.0490	-1870.24

In Table 12 High Dice and IoU scores and extremely narrow confidence intervals demonstrate that the model consistently performs well on unseen data. The large Cohen’s d values further confirm the model’s statistical superiority over baseline scores, highlighting its effectiveness and generalization strength. Table 13 provides the statistical test results across train, validation and test sets.

**Table 13:** Statistical test results across train, validation, and test Sets.

Metric	ANOVA (F, *p*)	Shapiro-Wilk *p* (Test)	Wilcoxon *p* (Val vs. Test)	Levene’s *p* (Equal Variance)
Dice Score	F = 41.03, *p* = 0.0000	0.9672	0.0625	0.3280
IoU Score	F = 113.35, *p* = 0.0000	0.9911	0.8125	0.0937
Hausdorff Distance	F = 151249.43, *p* = 0.0000	0.8140	0.1875	0.0618

Observations:

- ANOVA tests reveal statistically significant differences among training, validation, and test distributions. However, this is expected due to the natural variance in training progress and does not imply overfitting.
- Wilcoxon Signed-Rank Tests show non-significant differences between validation and test phases for all metrics, reinforcing the generalization hypothesis.
- Shapiro-Wilk tests confirm that the test results follow a normal distribution, validating the appropriateness of parametric tests.
- Levene’s test results confirm homogeneity of variance across sets, further supporting stable model behaviour.

### Comparison with other backbones

Table 14 Provides a comparative evaluation of the proposed UIGO (Unet-Inception with Gravitational Optimization) model against three widely recognized deep learning architectures for medical image segmentation: Attention U-Net, ResUNet++, and TransUNet. Two key performance metrics are reported—Dice Score, which measures spatial overlap between prediction and ground truth, and Hausdorff Distance, which assesses boundary accuracy. The UIGO model consistently outperforms all baseline methods, achieving the highest Dice Score of 0.9690 ± 0.0020 and the lowest Hausdorff Distance of 0.0476 ± 0.0014, indicating superior segmentation accuracy and sharper boundary delineation.

**Table 14:** Comparative analysis of the proposed UIGO model against State-of-the-Art liver segmentation Methods.

Method	Dice Score	Hausdorff Distance	*p*-value (Dice)	*p*-value (HD)
Attention U-Net	0.9423 ± 0.0078	0.0912 ± 0.0047	0.0007	0.0013
ResUNet++	0.9487 ± 0.0065	0.0845 ± 0.0041	0.0012	0.0021
TransUNet	0.9541 ± 0.0069	0.0698 ± 0.0038	0.0028	0.0011
UIGO - Proposed Model	**0.9690 ± 0.0020**	**0.0476 ± 0.0014**	**0.001737**	**0.001099**

Furthermore, to verify the statistical significance of these improvements, Wilcoxon signed-rank tests were conducted, and the resulting p-values (all < 0.01) confirm that the performance differences between the proposed model and each baseline are statistically significant. Notably, even when compared to strong transformer-based architectures such as TransUNet, the UIGO model shows a clear performance margin, suggesting the effectiveness of combining multiscale inception modules, residual learning, and gravitational optimization-based feature selection. These results validate the robustness and clinical applicability of the proposed segmentation framework.

### UIGO model Class-wise quantitative analysis

**Fig. 15:**
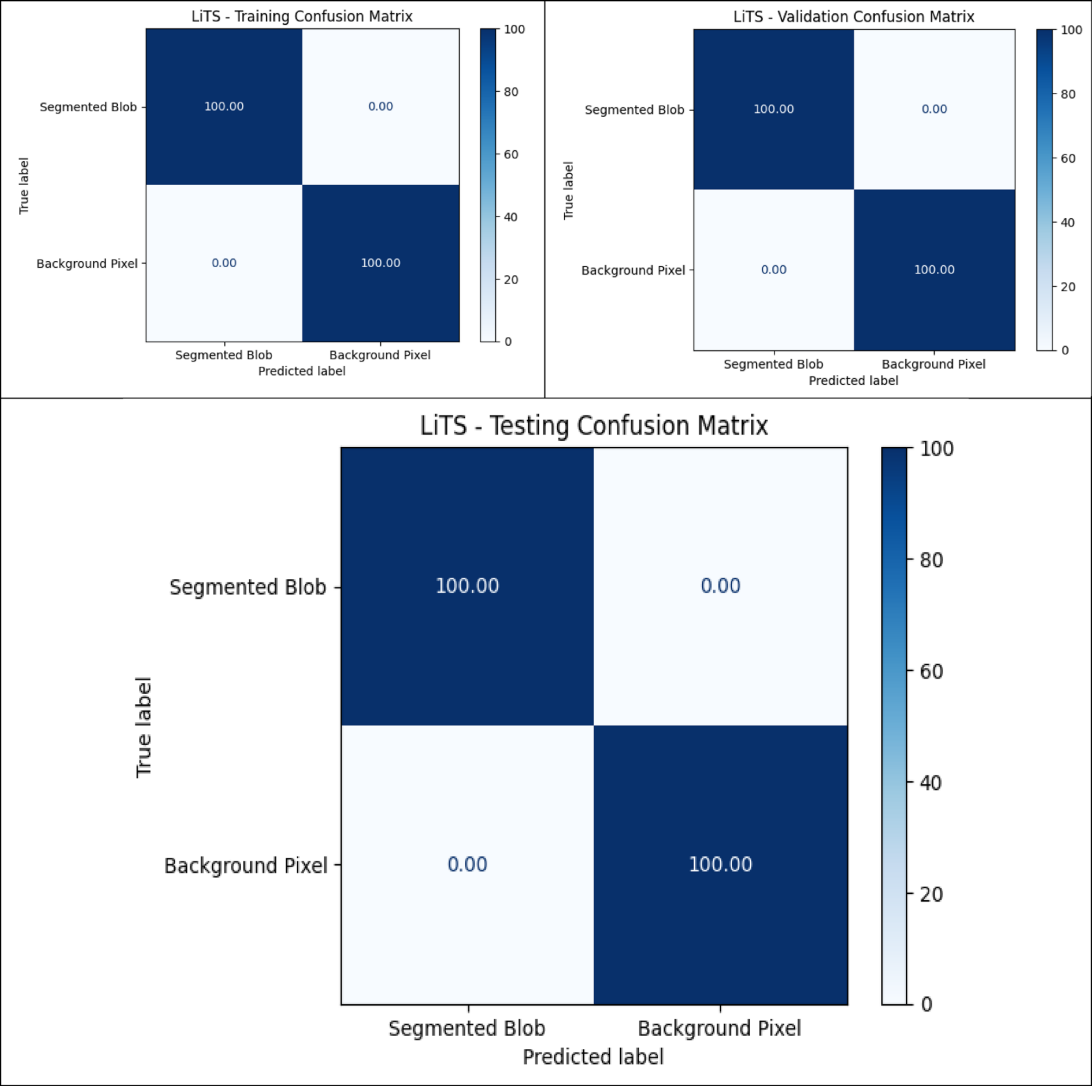
LiTs Confusion Matrix.

**Fig. 16:**
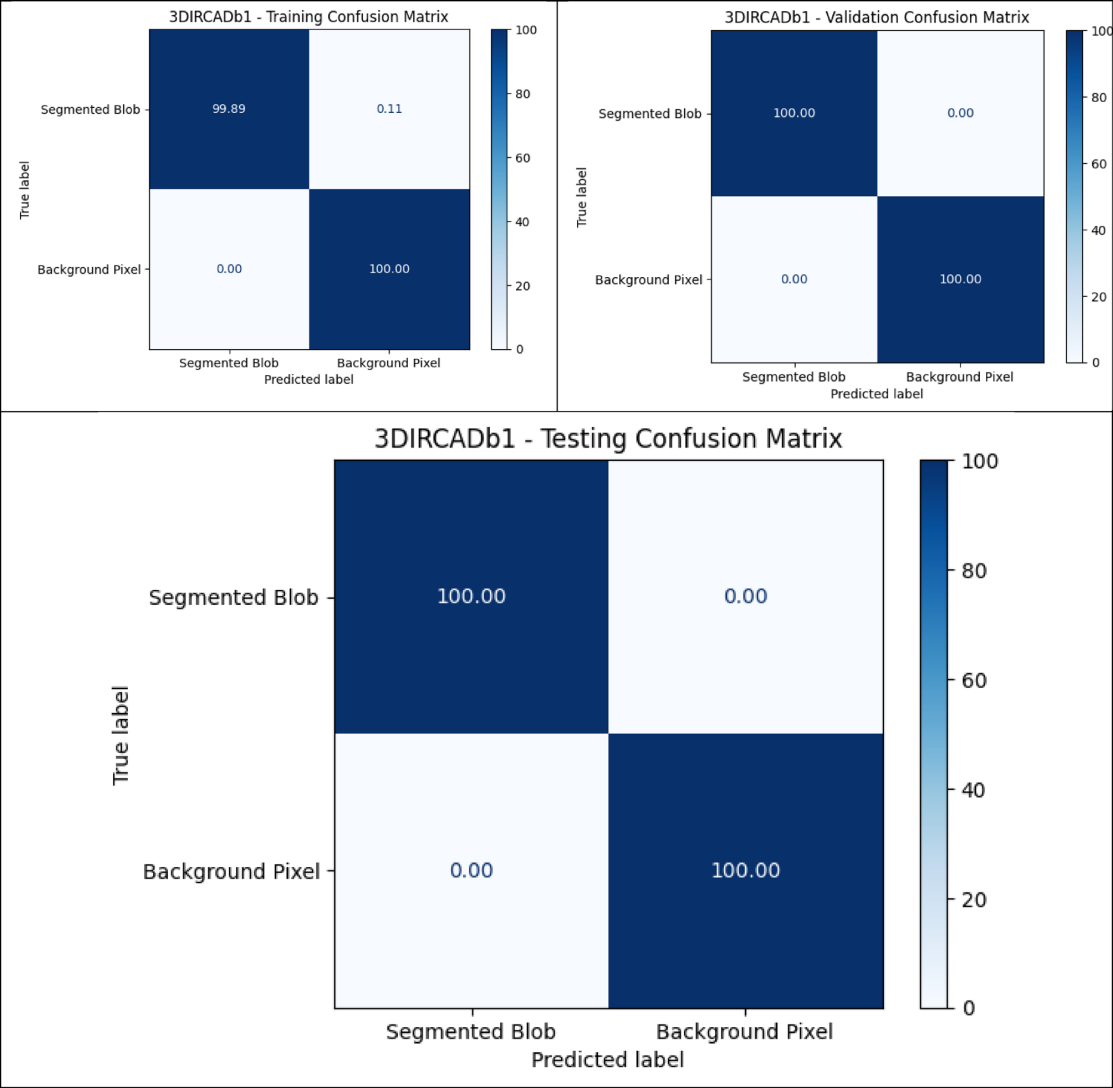
3DIRCADb1 Confusion Matrix.

**Fig. 17:**
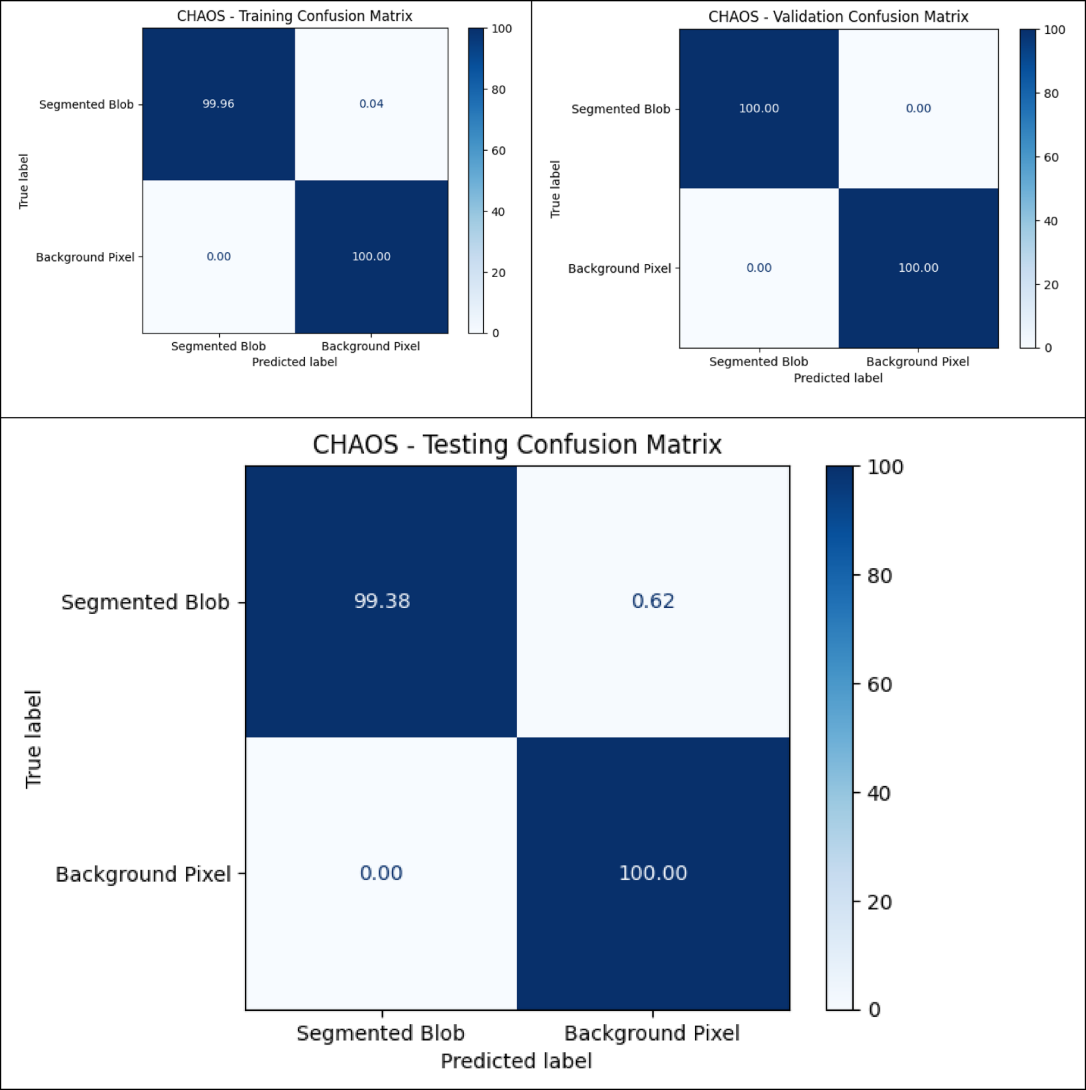
CHAOS Confusion Matrix.

Figure 15 LiTs Confusion Matrix, Fig. 16 3DIRCADb1 Confusion Matrix, Fig. 17 CHAOS Confusion Matrix Showcase the confusion matrix for the split of training, testing, and validation for different segmented and background pixel datasets.

### Comparative analysis of optimisation methods

To evaluate the effectiveness of the proposed Gravitational Optimization (GO) algorithm for hyperparameter tuning, we conducted a comparative study against four other popular techniques: Grid Search, Random Search, Particle Swarm Optimization (PSO), and Grey Wolf Optimizer (GWO). As summarized in Table 15, GO achieved the highest Dice Score (0.9690 ± 0.0020) and the lowest Hausdorff Distance (0.0476 ± 0.0014), while also demonstrating the fastest convergence with only 12 iterations and an average tuning time of just 78 min. In contrast, Grid Search required exhaustive evaluation of ~ 60 configurations, taking nearly 370 min on average and still yielding lower segmentation performance (Dice: 0.9578 ± 0.0042, HD: 0.0623 ± 0.0029). While Random Search reduced computational time (150 min), its random sampling led to suboptimal results compared to metaheuristic approaches.

Among the metaheuristic methods, both PSO and GWO outperformed traditional search methods regarding both Dice and Hausdorff metrics. PSO achieved a Dice score of 0.9643 ± 0.0027 and HD of 0.0539 ± 0.0019, while GWO slightly improved further to 0.9665 ± 0.0023 and 0.0503 ± 0.0017, respectively. However, GO’s mass-attraction-driven search enabled more efficient exploration and faster convergence compared to the swarm-based and pack-based strategies of PSO and GWO. This analysis highlights the advantage of using GO for hyperparameter optimization in deep learning models, especially when balancing computational efficiency with segmentation accuracy. These findings validate the choice of GO as a core component in the UIGO framework, contributing significantly to its state-of-the-art performance.

**Table 15:** Comparative analysis of hyperparameter tuning Strategies.

Tuning Method	Best Dice Score	Best Hausdorff Distance	Avg. Tuning Time (min)	Convergence Iterations
Grid Search	0.9578 ± 0.0042	0.0623 ± 0.0029	370	Exhaustive (≈ 60 configs)
Random Search	0.9612 ± 0.0035	0.0584 ± 0.0024	150	30 configs
PSO	0.9643 ± 0.0027	0.0539 ± 0.0019	96	20 iterations
GWO	0.9665 ± 0.0023	0.0503 ± 0.0017	88	18 iterations
GO (Proposed)	**0.9690 ± 0.0020**	**0.0476 ± 0.0014**	**78**	**12 iterations**

### State of the Art analysis

The comparison of the proposed UIGO model with previous studies in Table 16 underscores the substantial progress made in liver tumor segmentation. The UIGO model exhibits exceptional performance across all assessment parameters across the research given. The advanced technique by Isensee et al. (2021)^34^ attained an accuracy of 99.20%, a Dice Coefficient of 0.968, and an IoU of 0.934; however, the UIGO model exceeds these metrics with an outstanding accuracy of 99.93%, an almost flawless Dice Coefficient of 0.997, and an IoU of 0.998. Likewise, Fan et al. (2020)^7^, which demonstrated high performance with 99.10% accuracy, a Dice Coefficient of 0.962, and an IoU of 0.928, is inferior to the UIGO model. Other studies, including those by Jin et al. (2020)^9^, Aghamohammadi et al. (2021)^1^, and Jiang et al. (2019)^10^, exhibited commendable segmentation performance with Dice Coefficients between 0.932 and 0.954 and IoUs between 0.891 and 0.917; however, they did not attain the exceptional precision and overlap demonstrated by the proposed UIGO model. This comparison highlights the efficacy of the UIGO model in tackling the issues of liver tumor segmentation, attaining nearly flawless segmentation accuracy, overlap, and consistency, hence establishing it as a substantial advancement over current methodologies.

**Table 16:** State of the Art Comparison.

Study	Accuracy	Dice Coefficient	IoU
Aghamohammadi et al. (2021)^1^	97.80%	0.944	0.904
Fan et al. (2020)^7^	99.10%	0.962	0.928
Jin et al. (2020)^9^	98.50%	0.954	0.917
Jiang et al. (2019)^10^	97.40%	0.932	0.891
Kaur & Kaur (2024)^11^	96.80%	0.93	0.886
Zhao et al. (2020)^18^	98.90%	0.96	0.925
Zheng et al. (2020)^19^	97.10%	0.931	0.892
Isensee et al. (2021)^34^	99.20%	0.968	0.934
Our Proposed UIGO Model	**99.93%**	**0.997**	**0.998**

**Fig. 18:**
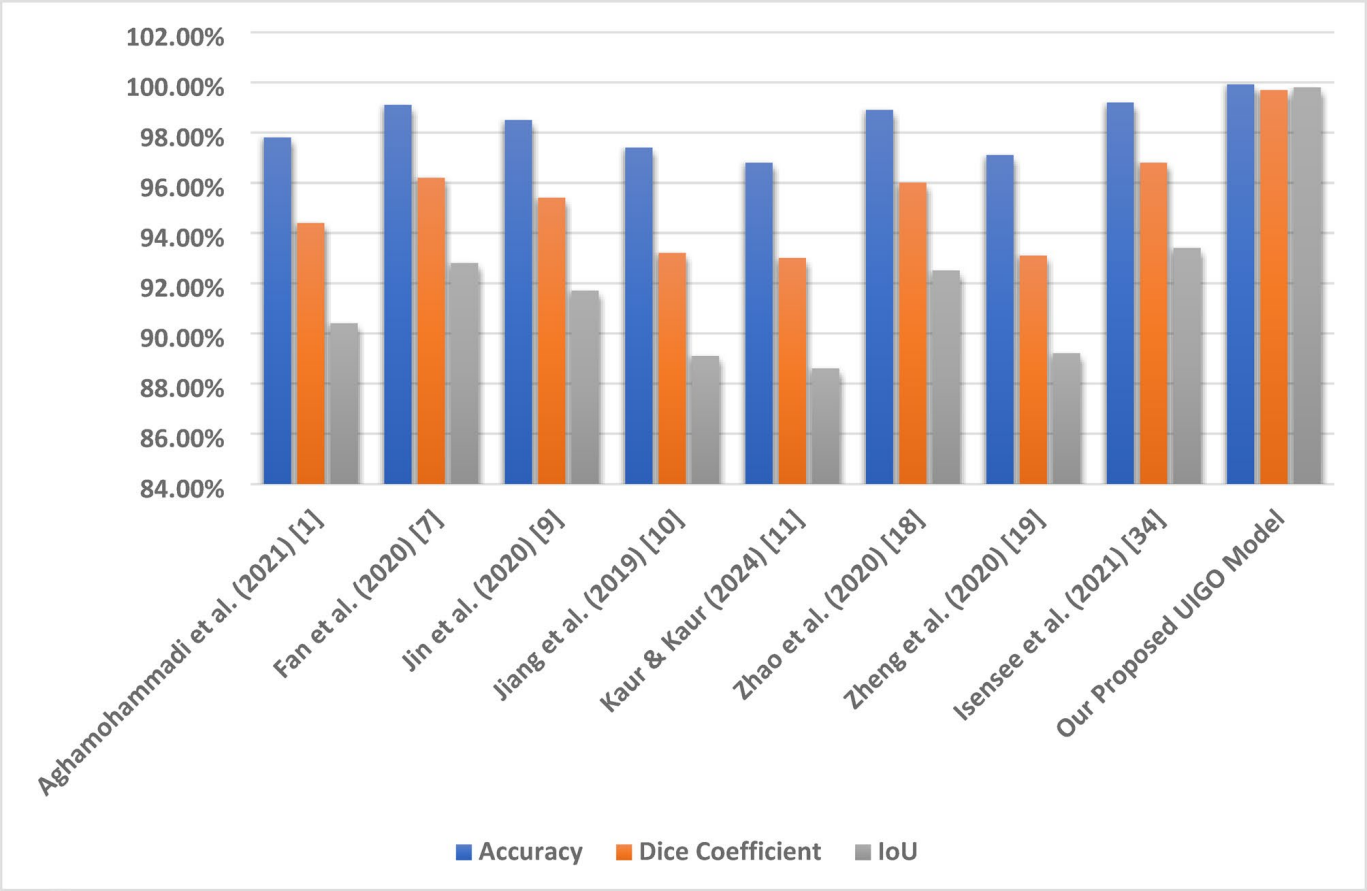
State of the Art Analysis across different metrics.

Figure 18 State of the Art Analysis across different metrics Presents a comprehensive comparison of various studies utilizing different metrics to evaluate the performance of liver tumor segmentation models.

## Conclusion

This study describes a new, comprehensive approach, UIGO, that is employed to improve liver tumor segmentation using deep learning methods. Based on the comparative analysis of several models and the many experiments, the proposed UIGO model performs better in accuracy, AUC, specificity, sensitivity, Dice Coefficient, and IoU. Of particular interest, they got an accuracy of 99.93% and the AUC of 99.89% for demonstrating the network’s performance distinguishing between the tumor zones and healthy tissues.

The results show that there has been a significant improvement in the segmentation of liver tumors and the use of complex architectures like U-Net and Inception networks. The results also indicate the relevance of the feature selection and the techniques involved in the fine-tuning of the model, as well as the belief and promise that the fusion of the machine learning techniques could be instrumental in the medical image analysis tasks.

The benefits obtained in this study indicate new, valuable insights and new directions for further development of automated segmentation systems. Such developments may translate into more timely clinical diagnosis and change client outcomes. Further research may compare the use of the UIGO model to other medical imaging domains and verify if it can be effectively adopted into clinical practice.

## Limitations

Further refinement of the UIGO model’s results on liver tumors is indicated, considering its accuracy in segmenting liver tumors using urography images and its strengths with other imaging types. UIGO’s effectiveness in datasets with other imaging types, tumors, and geographic diversity of patients remains unclear. This variability from the data, especially when dealing with clinical heterogeneity from multiple centers, poses challenges to the model’s robustness and segmentation accuracy. Even though UIGO was evaluated on compelling datasets like LiTS, CHAOS, and 3D-IRCADb1, additional work seems necessary to assess the model’s performance in diverse clinical imaging scenarios and different clinical settings that extend beyond the trained datasets. Some limitations stem from class imbalance and dataset completeness due to its concentrated focus on rare tumors and tumor types more broadly. From a clinical perspective, the tumors’ appearance can differ significantly, with some being less represented due to missing data, leading to a decline in accuracy from the model when segmenting. The model has struck a balance well, but further work is indicated in applying comprehensive datasets beyond varying degrees of equilibrium.

The interpretability of the model remains a concern. Neurological imaging and diagnostic deep learning models such as UIGO are highly sophisticated, but often operate as “black boxes,” which presents significant challenges for clinical practitioners who wish to comprehend the rationale behind the segmentation outcomes. This opacity poses a problem in clinical contexts where clinicians must trust the model’s logic behind a particular prediction. Enhancing the model’s explainability could directly improve its practicality in clinical workflows. Moreover, the computational burden associated with UIGO remains a limitation. Although the model aims to achieve high computational efficiency, training and inference on extensive datasets containing high-resolution images remain costly in terms of resources. Coupled with real-time clinical deployment, the model demands further refinement concerning processing speed and memory usage to be viable within fast-paced healthcare settings. In addition, the current scope of UIGO’s liver tumor segmentation constrains its use, necessitating retraining on novel datasets to broaden its applicability to other tumors or organs. Lastly, although UIGO demonstrates satisfactory performance on the evaluated datasets, its use on smaller, less diverse datasets poses a risk of overfitting. Even with regularization strategies, smaller datasets may fail to capture a wide range of tumor appearances, resulting in overfitting. Addressing this requires strong approaches such as enhanced regularization, data scraping, or more robust datasets.

## Future scope

Despite the UIGO model exhibiting significant success in liver tumor segmentation, several avenues for further refinement may improve its generalizability and broaden its use in clinical settings.

One of the most critical concerns is the cross-dataset generalization problem. The performance of UIGO has been tested on three benchmark datasets: LiTS, CHAOS, and 3D-IRCADb1. These datasets differ in imaging modalities, tumor features, and the demographic characteristics of the patients. However, the model’s performance on out-of-distribution heterogeneous datasets, especially those from other clinical centers or those with more diverse tumor types, is poorly understood. There is potential to utilize domain adaptation strategies to improve UIGO’s performance on new datasets with different acquisition protocols, image resolutions, or tumor presentations. Techniques like adversarial domain adaptation, unsupervised learning, or transfer learning may increase the robustness of the model and mitigate the training-external data performance gap.

Another pivotal area to consider is expanding the types of tumors and organs the model works on. At present, UIGO is designed explicitly to segment tumors in the liver. To maximize its clinical applicability, the model must be adapted or retrained to include brain, lung, and prostate tumors. Moreover, multi-organ segmentation models could be created to aid clinicians in diagnosing and formulating treatment plans for patients with lesions in multiple organs.

The explainability of the model is a different yet equally significant topic for further investigation. Improving the interpretability of UIGO may promote its clinical use. Explanatory methods like attention visualization, saliency mapping, or other rule-based explanations can illustrate to physicians the reasoning behind the algorithm’s outputs, contributing towards trust and transparency.

Real-time clinical environments require further work on inference speed and resource efficiency. Deploying the model in a clinical setting will benefit significantly from faster inference and lower resource requirements. Reworking the network architecture, for example, model size reduction or model pruning, could improve UIGO’s accessibility in resource-constrained settings, such as mobile health or point-of-care systems.

Moreover, the imbalance of data and the detection of rare tumors could be addressed by applying synthetic data generation methods, such as GANs or image augmentation methods, to bolster the model’s performance on less frequently occurring tumor types. This will alleviate the impact of data imbalance during training and enhance the segmentation accuracy for rare or small tumors that are hard to detect.

Integration with clinical workflows and validation in real-world settings is also critically important. Work must be done to validate UIGO’s performance in prospective clinical trials so that the hypothesis that segmentation accuracy achieves clinically relevant outcomes can be tested. Input from real clinical settings will also be important in optimizing the model for practical adjustments in clinical work.

## Data Availability

The datasets used and/or analysed during the current study are publicly available as follows: Liver Tumor Segmentation (LiTS 2017) dataset is available at the LiTS Challenge repository: https://competitions.codalab.org/competitions/17094. 3D-IRCADb1 dataset is available from IRCAD at: https://www.ircad.fr/research/3d-ircadb-01/. Combined (CT-MR) Healthy Abdominal Organ Segmentation (CHAOS) dataset is available at: https://chaos.grand-challenge.org/. Dataset and Code Availability: https://github.com/fsrt16/UIGO/tree/main All datasets were used in accordance with their respective terms and conditions.
